# Clinical outcomes in brief psychotic episodes: a systematic review and meta-analysis

**DOI:** 10.1017/S2045796021000548

**Published:** 2021-11-04

**Authors:** U. Provenzani, G. Salazar de Pablo, M. Arribas, F. Pillmann, P. Fusar-Poli

**Affiliations:** 1 Department of Brain and Behavioural Sciences, University of Pavia, Pavia, Italy; 2 Early Psychosis: Interventions and Clinical-detection (EPIC) Lab, Department of Psychosis Studies, Institute of Psychiatry, Psychology & Neuroscience, King's College London, London, UK; 3 AWO Center of Psychatry, Halle, Germany; 4 Martin Luther University Halle-Wittenberg, Halle, Germany; 5 Outreach and Support in South London (OASIS) service, South London and Maudsley NHS Foundation Trust, London, UK

**Keywords:** ATPD, BPD, brief psychotic episode, psychosis

## Abstract

**Aims:**

Patients with brief psychotic episodes (BPE) have variable and fluctuating clinical outcomes which challenge psychiatric care. Our meta-analysis aims at providing a comprehensive summary of several clinical outcomes in this patient group.

**Methods:**

A multistep systematic PRISMA/MOOSE-compliant literature search was performed for articles published from inception until 1st March 2021. Web of Science database was searched, complemented by manual search of original articles reporting relevant outcomes (psychotic recurrence, prospective diagnostic change or stability, remission, quality of life, functional status, mortality and their predictors) for patients diagnosed with acute and transient psychotic disorders (ATPD), brief psychotic disorders (BPD), brief intermittent psychotic symptoms (BIPS) and brief limited intermittent psychotic symptoms (BLIPS). Random-effects methods and *Q*-statistics were employed, quality assessment with Newcastle-Ottawa Scale, assessment of heterogeneity with *I*^2^ index, sensitivity analyses (acute polymorphic psychotic disorders, APPD) and multiple meta-regressions, assessment of publication bias with funnel plot, Egger's test and meta-regression (psychotic recurrence and sample size).

**Results:**

A total of 91 independent articles (*n* = 94 samples) encompassed 37 ATPD, 24 BPD, 19 BLIPS and 14 BIPS samples, totalling 15 729 individuals (mean age: 30.89 ± 7.33 years, mean female ratio: 60%, 59% conducted in Europe). Meta-analytical risk of psychotic recurrence for all BPE increased from 15% (95% confidence interval (CI) 12–18) at 6 months, 25% (95% CI 22–30) at 12 months, 30% (95% CI 27–33) at 24 months and 33% (95% CI 30–37) at ⩾36 months follow-up, with no differences between ATPD, BPD, BLIPS and BIPS after 2 years of follow-up. Across all BPE, meta-analytical proportion of prospective diagnostic stability (average follow-up 47 months) was 49% (95% CI 42–56); meta-analytical proportion of diagnostic change (average follow-up 47 months) to schizophrenia spectrum psychoses was 19% (95% CI 16–23), affective spectrum psychoses 5% (95% CI 3–7), other psychotic disorders 7% (95% CI 5–9) and other (non-psychotic) mental disorders 14% (95% CI 11–17). Prospective diagnostic change within APPD without symptoms of schizophrenia was 34% (95% CI 24–46) at a mean follow-up of 51 months: 18% (95% CI 11–30) for schizophrenia spectrum psychoses and 17% (95% CI 10–26) for other (non-psychotic) mental disorders. Meta-analytical proportion of baseline employment was 48% (95% CI 38–58), whereas there were not enough data to explore the other outcomes. Heterogeneity was high; female ratio and study quality were negatively and positively associated with risk of psychotic recurrence, respectively. There were no consistent factor predicting clinical outcomes.

**Conclusions:**

Short-lived psychotic episodes are associated with a high risk of psychotic recurrences, in particular schizophrenia spectrum disorders. Other clinical outcomes remain relatively underinvestigated. There are no consistent prognostic/predictive factors.

## Introduction

Since the 19th century, brief psychotic episodes (BPE) have been characterised as a divergent and shifting nosographic concept, providing a major challenge both for clinical practice and classification. Historically, their fleeting and dynamical nature constituted an early anomaly within the Kraepelinian dichotomous concept of psychoses (dementia praecox *v.* manic-depressive illness; Kraepelin, 1899). The historical development of operationalisations for BPE has been previously appraised (Fusar-Poli *et al*., 2016a) by our group. Briefly, World Health Organization includes the concept of acute and transient psychotic disorders (ATPD) in ICD-10 within the group of ‘schizophrenia, schizotypal and delusional disorders’ (World Health Organization, 1992), with six subtypes, including disorders defined by acute onset of frank psychotic symptoms (acute within 2 weeks; abrupt within 48 h) and complete recovery within 1–3 months even with antipsychotic treatment. However, the 11th edition of the ICD has narrowed the ATPD category to the subtype of acute polymorphic psychotic disorders (APPD) without the symptoms of schizophrenia (Castagnini and Foldager, 2014). DSM-5 includes brief psychotic disorder (BPD) within the ‘schizophrenia spectrum and other psychotic disorders’, defined by the presence of at least one across four of five core symptoms of schizophrenia (negative symptoms are not included): delusions, hallucinations, disorganised speech and grossly disorganised or catatonic behaviour (American Psychiatric Association, 2013). Duration of the episode must be less than 1 month and sudden onset and polymorphic and fluctuating nature of symptoms are not mandatory criteria (Gaebel, 2012). The paradigm of clinical high-risk states of psychosis (CHR-P) operationalised BPE not as frank psychotic disorder, but as a state of increased clinical risk for developing subsequent persistent psychotic disorders. Brief limited intermittent psychotic symptoms (BLIPS) (Yung *et al*., 1996) last less than 7 days and accommodate potential ‘transdiagnostic’ (Fusar-Poli, 2019; Fusar-Poli *et al*., 2019c) comorbidities. Brief intermittent psychotic symptoms (BIPS) exclude individuals with seriously disorganising and dangerous features (Miller *et al*., 2003) and extend the duration of brief psychotic symptoms up to 3 months.

Given these differences, in previous studies we employed meta-analyses to test the prognostic significance of these competing operationalisations. We found no difference in their risk of psychotic recurrence at any follow-up timepoint, but a lower risk of recurrence compared to first-episode remitting schizophrenia (Fusar-Poli *et al*., 2016a). We also demonstrated meta-analytically that, at 4.5 years follow-up, about 56% of patients with BPE retained their index diagnosis (prospective diagnostic stability) whereas 44% shifted to other diagnoses (prospective diagnostic change), schizophrenia being the most frequent one (21%, 95% confidence interval (CI) 16–25) (Fusar-Poli *et al*., 2016c). Other clinical outcomes such as remission, quality of life, vocational status and mortality have traditionally seen as more favourable than in schizophrenia (Pillmann and Marneros, 2005) but findings have been conflicting. Similarly, predictors of these outcomes appear patchy and inconsistent and require a systematic appraisal. These meta-analytic summaries had been completed more than 5 years ago, and several recent publications have emerged, which require periodical updates of the evidence. Our aim was to provide an updated systematic review and meta-analysis which comprehensively addresses for the first time multiple clinical outcomes in BPE encompassing psychotic recurrence, prospective diagnostic stability and change, remission, quality of life, functional/vocational status and mortality and systematically appraise the consistency of predictors of these outcomes.

## Methods

This study (protocol registered on https://osf.io/5dw98) was conducted in accordance with the Preferred Reporting Items for Systematic reviews and Meta-Analyses (Moher *et al*., 2009) and the Meta-analysis Of Observational Studies in Epidemiology (Stroup *et al*., 2000) (online Supplementary eTables 1 and 2).

### Search strategy and selection criteria

A multistep systematic PRISMA-compliant literature search was performed for articles published from inception until 1st March 2021 (see the details in online Supplementary eMethods 1). The references of systematic reviews or meta-analyses that were retrieved during the systematic literature search were manually searched. Articles identified were screened as abstracts. The remaining articles were assessed for inclusion eligibility against the inclusion and exclusion criteria and decisions were made regarding their final inclusion in the meta-analysis.

The inclusion criteria were: (a) original studies, published in English; (b) conducted in individuals meeting BIPS or BLIPS criteria according to Structured Interview for Psychosis-Risk Syndromes (SIPS any version) (McGlashan TH, 2010) or Comprehensive Assessment of At-Risk Mental States (CAARMS any version) (Yung *et al*., 2006); ATPD according to ICD (any version) criteria (World Health Organization, 1992); or BPD according to DSM (any version) criteria (American Psychiatric Association, 2013) and (c) reporting on at least one of meta-analytic outcomes (see below).

The exclusion criteria were: (a) reviews, conference proceedings or pilot data sets; (b) unpublished data; (c) studies evaluating BPE but using criteria different from those established above; (d) studies that did not report specific information on the ATPD/BPD/BIPS/BLIPS group alone, but reported composite results including other subgroups (e.g. attenuated psychosis syndrome, APS and genetic risk and deterioration syndrome, GRD) and (e) studies with samples of individuals with other persisting psychotic disorders (e.g. schizophrenia). For the meta-analyses we additionally excluded studies with overlapping datasets, selecting the largest and most recent data set. Disagreements in selection criteria were resolved through discussion and consensus with a senior researcher.

### Outcome measures and data extraction

Two independent researchers extracted data. The primary outcome was risk of psychotic recurrence after at least 3 months since the index BPE. Secondary outcomes were the prospective diagnostic stability (defined as the proportion of baseline patients retaining the same ICD/DSM psychotic diagnosis over time) (Fusar-Poli *et al*., 2016c) or diagnostic change to any other mental disorder (defined as the proportion of baseline patients shifting to other ICD/DSM diagnoses at follow-up) (Fusar-Poli *et al*., 2016c), remission (defined as indicated in each study), quality of life (defined as indicated in each study, at baseline and/or follow-up), functional/vocational status (defined as indicated in each study at baseline and/or follow-up), mortality (number of patients deceased during the follow-up) or predictors of these outcomes (any regression models testing the association between factors and clinical outcomes, within longitudinal designs). When the predictors were not directly presented in the text, they were extracted from associated information (e.g. graphs); corresponding authors were also contacted to retrieve missing data. From each study, a predetermined set of variables characterising the study, outcomes or predictors was included (online Supplementary eMethods 2). Data were extracted closest to the follow-up time of interest. Disagreements in selection/extraction criteria were resolved through discussion and consensus with a senior researcher.

### Quality assessment

The quality of the included studies was evaluated using a modified version of the Newcastle-Ottawa Scale (NOS) for cohort studies, which has been repeatedly used (Catalan *et al*., 2020). Studies were awarded a maximum of eight points on items related to the representativeness, exposure, outcomes, follow-up period and loss to follow-up.

### Strategy for data synthesis

Meta-analyses were attempted when there were enough data for each outcome, otherwise outcomes were systematically described.

The primary meta-analytical outcome was risk of psychotic recurrence across BPE. This was estimated by extracting the raw counts of psychotic recurrence in individuals with an initial short BPE (defined according to the inclusion criteria (b)) at 6 months (including a 3–9 months range); 12 months (including a 10–18 months range); 24 months (including a 19–29 months range); 36 months (including ⩾30 months) follow-up. The primary effect size was the meta-analytical proportion of psychotic recurrence. Because the studies were expected to be heterogenous, random-effects models were used. Heterogeneity among study point estimates was assessed with the *Q*-statistics, with the proportion of the total variability in the effect size estimates being evaluated with the *I*^2^ index (Lipsey and Wilson, 2001) which does not depend upon the number of studies included. The meta-analysis was stratified by operationalisations of BPE: BIPS, BLIPS, ATPD or BPD. The four main operationalisations (BIPS, BLIPS, ATPD and BPD) were compared testing for between-group heterogeneity (*Q*). Sensitivity analyses, removing one study at a time, were conducted to test the robustness of results. Publication bias was assessed with funnel plot inspection, Egger's test and meta-regression between the main outcome (psychotic recurrence) as dependent variable and sample size as independent variable.

Planned meta-analyses on secondary outcomes included: (i) prospective diagnostic stability (effect size: proportion), (ii) prospective diagnostic change to any other mental disorder (effect size: proportion), (iii) remission (effect size: standardised mean difference), (iv) quality of life (effect size: standardised mean difference) at baseline and/or follow-up, (v) functional/vocational status (effect size: proportion) at baseline and/or follow-up and (vi) mortality (effect size: proportion). The analyses (i–ii) were not conducted for BLIPS/BIPS because these do not correspond to standard ICD/DSM diagnoses at baseline and were conducted pooling all available studies and estimating the average follow-up time. Within analyses (i–ii), we additionally clustered the individual ICD/DSM diagnoses across NICE-compliant diagnostic spectra (National Institute for Health and Care Excellence, 2014): schizophrenia spectrum psychoses (schizophrenia, schizophreniform disorder and schizoaffective disorder), affective spectrum psychoses (mania with psychosis and/or bipolar disorder with psychosis and/or depression with psychosis), other psychotic disorders and other (non-psychotic) mental disorders. For all outcomes (primary and secondary), we also sought to perform additional analyses restricted to the ATPD subtype which will be retained in the ICD-11 version (APPD without symptoms of schizophrenia). Sensitivity analyses were planned on the primary outcome, by excluding one study per time and rerunning the analyses. We performed multiple meta-regression analyses (i.e. employing two meta-regressor factors at the same time) of factors that were known to moderate transition risk when at least ten studies were available. The fixed meta-regressor factor was follow-up time, which was combined with: mean age, female ratio, publication year and study quality (NOS score). Comprehensive Meta-Analysis Software v3.0 was used for the analyses (Borenstein *et al*., 2013).

## Results

### Database

**Fig. 1.**
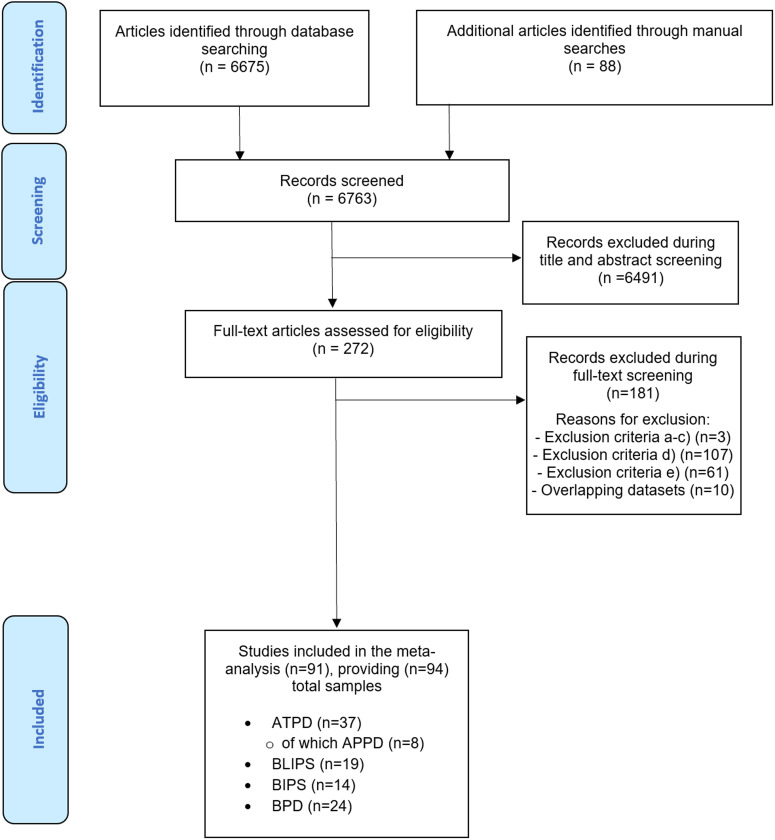
PRISMA 2009 flow diagram.

The systematic literature search (PRISMA flow-chart, Fig. 1) identified 91 independent articles, some of which contributed with more than one sample. The total number of samples (*n* = 94) included 37 ATPD, 24 BPD, 19 BLIPS and 14 BIPS samples. Eight samples (1262 patients) comprising ATPD subjects also provided data for APPD without symptoms of schizophrenia subtype. The total database included 15 729 individuals, with a mean age of 30.89 ± 7.33 years and an average female ratio of 60.0% (range from 8.3 to 86.2%). In total, 59% of the studies were conducted in Europe, 28% in Asia, 7% in North America, 3% in Australia, 2% in Africa and 1% in South America. The characteristics of the studies included in the meta-analyses are illustrated in online Supplementary eTable 3.

### Risk of psychotic recurrence of BPE

**Fig. 2.**
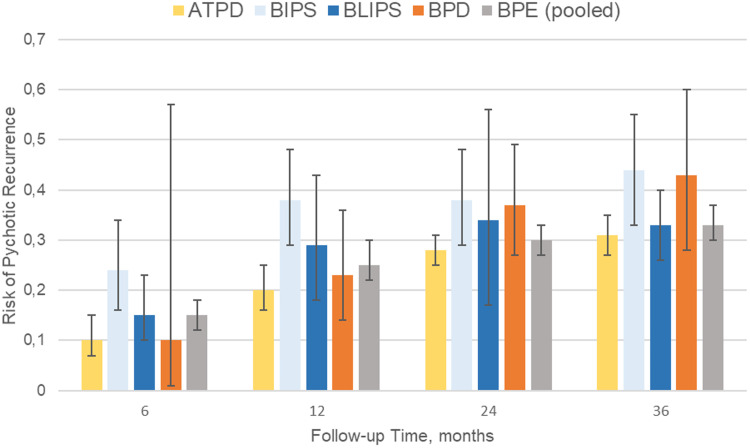
Meta-analysis of risk of psychotic recurrences in BPE. ATPD, acute and transient psychotic disorder; BIPS, brief intermittent psychotic symptoms; BLIPS, brief limited intermittent psychotic symptoms; BPD, brief psychotic disorder.

Ninety-one independent samples reported primary outcome data at different follow-up time points (Table 1 and Fig. 2). The meta-analytical risk of psychotic recurrence for all BPE was 15% (95% CI 12–18) at 6 months, 25% (95% CI 22–30) at 12 months, 30% (95% CI 27–33) at 24 months and 33% (95% CI 30–37) at ⩾36 months follow-up. The meta-analytical risk of psychotic recurrence for ATPD was 10% (95% CI 7–15) at 6 months, 20% (95% CI 16–25) at 12 months, 28% (95% CI 25–31) at 24 months and 31% (95% CI 27–35) at ⩾36 months follow-up; for BIPS, 24% (95% CI 16–34) at 6 months, 38% (95% CI 29–48) at 12 months, 38% (95% CI 29–48) at 24 months and 44% (95% CI 33–55) at ⩾36 months follow-up; for BLIPS, 15% (95% CI 10–23) at 6 months, 29% (95% CI 18–43) at 12 months, 34% (95% CI 17–56) at 24 months and 33% (95% CI 26–40) at ⩾36 months follow-up; for BPD, 10% (95% CI 1–57) at 6 months, 23% (95% CI 14–36) at 12 months, 37% (95% CI 27–49) at 24 months and 43% (95% CI 30–37) at ⩾36 months follow-up. There were significant between-group differences at 6 months (*p* = 0.020) and 12 months (*p* = 0.003), mostly driven by the BIPS group, but not at 24 (*p* = 0.110) and at ⩾36 (*p* = 0.100) months (Table 1). The risk of psychotic recurrence in APPD without symptoms of schizophrenia was 21% (95% CI 14–31) at mean follow-up of 51 months (online Supplementary eFig. 1).

**Table 1.** Meta-analysis of risk of psychotic recurrences in BPE

	Follow-up, months			
Characteristic	6	12	24	⩾36
No. of samples	22	35	31	40
No. of patients analysed	3874	4559	4189	13 624
Risk of psychotic recurrence, mean (95% CI)				
ATPD	0.10 (0.07–0.15)	0.20 (0.16–0.25)	0.28 (0.25–0.31)	0.31 (0.27–0.35)
BIPS	0.24 (0.16–0.34)	0.38 (0.29–0.48)	0.38 (0.29–0.48)	0.44 (0.33–0.55)
BLIPS	0.15 (0.10–0.23)	0.29 (0.18–0.43)	0.34 (0.17–0.56)	0.33 (0.26–0.40)
BPD	0.10 (0.01–0.57)	0.23 (0.14–0.36)	0.37 (0.27–0.49)	0.43 (0.28–0.60)
BPE (pooled)	0.15 (0.12–0.18)	0.25 (0.22–0.30)	0.30 (0.27–0.33)	0.33 (0.30–0.37)
ATPD *v.* BIPS *v.* BLIPS *v.* BPD test for between-group heterogeneity (*Q*)	9.80	13.70	6.10	6.17
*p* value	0.02	<0.01	0.11	0.10
*I*^2^ (%)	67.78	66.67	43.81	84.55

ATPD, acute and transient psychotic disorder; BIPS, brief intermittent psychotic symptoms; BLIPS, brief limited intermittent psychotic symptoms; BPD, brief psychotic disorder; BPE, brief psychotic episodes.

### Proportion of prospective diagnostic stability and diagnostic change

**Fig. 3.**
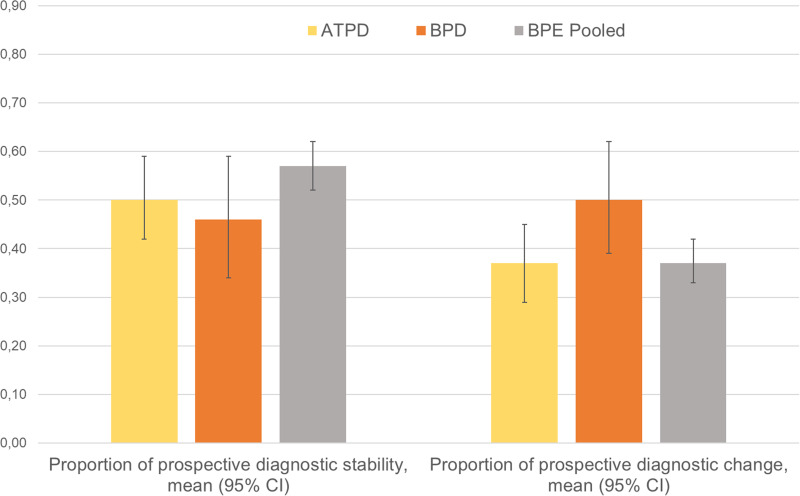
Meta-analysis of prospective diagnostic stability and change of BPE. ATPD, acute and transient psychotic disorder; BPD, brief psychotic disorder *Note*: Diagnostic stability and diagnostic change values are not reciprocal, because they were calculated analysing different datasets.

Sixty-one independent samples (15 034 patients) reported prospective diagnostic stability or diagnostic change data at different follow-up time points (Table 2). The meta-analytical proportion of prospective diagnostic stability (average follow-up 47 months) was 49% (95% CI 42–56) for all BPE. It was 50% (95% CI 42–59) for ATPD and 46% (95% CI 34–59) for BPD (Table 2 and Fig. 3), with no significant between-group differences (*p* = 0.59).

**Table 2.** Prospective diagnostic stability and change of BPE

	Proportion of prospective diagnostic stability, mean (95% CI)	Proportion of prospective diagnostic change, mean (95% CI)
ATPD	0.50 (0.42–0.59)	0.37 (0.29–0.45)
BPD	0.46 (0.34–0.59)	0.50 (0.39–0.62)
BPE (pooled)	0.49 (0.42–0.56)	0.41 (0.35–0.48)
ATPD *v.* BPD test for between-group heterogeneity (*Q*)	0.30	3.47
*p* value	0.59	0.06
*I*^2^	97.33	97.17

ATPD, acute and transient psychotic disorder; BPD, brief psychotic disorder; BPE, brief psychotic episodes.

The meta-analytical proportion of prospective diagnostic change (average follow-up 47 months) was 41% (95% CI 35–48) for all BPE: 37% (95% CI 29–45) for ATPD and 50% (95% CI 39–62) for BPD. There were no significant between-group differences (*p* = 0.063). Specifically, the meta-analytical proportion of diagnostic change to schizophrenia spectrum psychoses was 19% (95% CI 16–23) for all BPE: 19% (95% CI 15–24) for ATPD, 20% (95% CI 14–28) for BPD (between-group difference *p* = 0.770); to affective spectrum psychoses: 5% (95% CI 3–7) for all BPE, 1% (95% CI 0.6–2) for ATPD, 10% (95% CI 6–15) for BPD (between-group difference *p* < 0.01); to other psychotic disorders: 7% (95% CI 5–9) for all BPE, 3% (95% CI 2–5) for ATPD, 12% (95% CI 8–16) for BPD (between-group difference *p* < 0.010); to other (non-psychotic) mental disorders: 14% (95% CI 11–17) for all BPE, 14% (95% CI 12–18) for ATPD, 14% (95% CI 8–23) for BPD (between-group difference *p* = 0.950).

The meta-analytical prospective diagnostic stability for APPD without symptoms of schizophrenia (average follow-up 51 months) was 56% (95% CI 46–66), prospective diagnostic change was 34% (95% CI 24–46), proportion of diagnostic change to schizophrenia spectrum psychoses was 18% (95% CI 11–30) and of other (non-psychotic) mental disorders was 17% (10–26%) (online Supplementary eFig. 1). There were not enough data to meta-analyse change from APPD to affective spectrum psychoses or to other psychotic disorders.

### Remission

There were not enough data to meta-analyse this outcome. Four studies (two BPD and two ATPD samples) (Rahm and Cullberg, 2007; Castagnini *et al*., 2008; Castro-Fornieles *et al*., 2011; Bjorkenstam *et al*., 2013) reported proportion of remitted patients, defined as not having any mental disorder at follow-up, ranging from 14 (Castagnini *et al*., 2008) to 67% (Rahm and Cullberg, 2007).

### Quality of life

There were not enough data to meta-analyse this outcome. Two studies reported outcomes related to quality of life. One study (Aadamsoo *et al*., 2011) found that the World Health Organization Quality of Life scale (WHO-QoL) scores were not significantly different between ATPD and schizophrenia at baseline, but it became better in ATPD at follow-up. Another study (Satghare *et al*., 2020) reported that BPD, but not schizophrenia, is positively correlated with WHO-QoL scores at baseline.

### Functional/vocational status

Twelve samples (907 patients) (nine ATPD, two BLIPS, one BPD) reported the functional/vocational status. The meta-analytical proportion of patients with BPE who were employed at baseline was 48% (95% CI 38–58). There were not enough data to meta-analyse this outcome at follow-up.

### Mortality

There were not enough data to meta-analyse this outcome. Three studies (Castagnini *et al*., 2008, 2013a; Korner *et al*., 2009), all including ATPD samples, independently reported a proportion of deaths of 17.0% at 60 months follow-up (Castagnini *et al*., 2008), 5.6% at 84 months (Castagnini *et al*., 2013a) and 33.5% at 96 months (Korner *et al*., 2009).

### Predictors of clinical outcomes

Ten studies reported predictors for psychotic recurrence in BPE (Table 3). No factor was consistently associated with psychotic recurrence. Predictors of diagnostic stability partially overlap (but not consistently) with predictors of recurrence, with studies suggesting that older age at onset (Aadamsoo *et al*., 2011; Queirazza *et al*., 2014; Poon and Leung, 2017; Wang *et al*., 2018), female gender (Singh *et al*., 2004; Castagnini *et al*., 2013b; Castagnini and Foldager, 2014; Queirazza *et al*., 2014; Wang *et al*., 2018), abrupt onset (Sajith *et al*., 2002; Rusaka and Rancans, 2014a), seriously disorganising or dangerous symptoms (Fusar-Poli *et al*., 2017a), polymorphic symptomatology and absence of schizophrenic features at onset (Sajith *et al*., 2002; Aadamsoo *et al*., 2011; Salvatore *et al*., 2011; Castagnini *et al*., 2013b; Rusaka and Rancans, 2014a, 2014b) predict higher diagnostic stability (Lopez-Diaz *et al*., 2020). One study suggested that female gender was associated with early remission (Esan and Fawole, 2014). There were no predictors of quality of life, functional/vocational status or mortality.

**Table 3.** Predictors of psychotic recurrence in BPE (ATPD/BPD/BLIPS/BIPS)

Predictor	Association with psychotic recurrence
Acute onset	↓(Rusaka and Rancans, 2014b) ↔ (Rusaka and Rancans, 2014b; Fusar-Poli *et al*., 2019a, 2019b, 2019c)
Acute stress	↔(Fusar-Poli *et al*., 2019a, 2019b, 2019c)
Affective disturbances	↔(Rusaka and Rancans, 2014a, 2014b)
Antipsychotic dosage	↑(Wang *et al*., 2018)
Anxiety	↑ (Rusaka and Rancans, 2014a); ↔(Suda *et al*., 2005; Rusaka and Rancans, 2014b)
Delusional ideation	↔(Suda *et al*., 2005; Rusaka and Rancans, 2014a, 2014b)
Depressed mood	↔(Suda *et al*., 2005; Rusaka and Rancans, 2014a, 2014b)
Duration of psychotic symptoms	↔(Fusar-Poli *et al*., 2017a, 2019a, 2019b, 2019c; Wang *et al*., 2018)
Duration/number of hospitalisations over follow-up	↑(Aadamsoo *et al*., 2011; Queirazza *et al*., 2014; Poon and Leung, 2017; Wang *et al*., 2018); ↔(Suda *et al*., 2005; Rusaka and Rancans, 2014b)
Employment	↔(Aadamsoo *et al*., 2011; Fusar-Poli *et al*., 2017a)
Ethnicity	↔(Fusar-Poli *et al*., 2017a)
Family history of mental disorders	↑(Wang *et al*., 2018); ↔(Suda *et al*., 2005; Abe *et al*., 2006; Rusaka and Rancans, 2014b; Poon and Leung, 2017)
Frequency of episodes over follow-up	↑(Fusar-Poli *et al*., 2017a); ↔ (Suda *et al*., 2005)
Hallucinations	↑(Rusaka and Rancans, 2014a); ↔ (Suda *et al*., 2005; Rusaka and Rancans, 2014b)
Hazardousness	↔(Aadamsoo *et al*., 2011)
Illicit substances	↔(Fusar-Poli *et al*., 2019a, 2019b, 2019c)
Irritability or mood elation	↔(Suda *et al*., 2005)
Level of functioning	↔(Aadamsoo *et al*., 2011; Fusar-Poli *et al*., 2017a)
Life events	↔(Suda *et al*., 2005; Abe *et al*., 2006)
Male gender	↑(Queirazza *et al*., 2014; Wang *et al*., 2018); ↔(Suda *et al*., 2005; Abe *et al*., 2006; Aadamsoo *et al*., 2011; Fusar-Poli *et al*., 2017a; Poon and Leung, 2017)
Marital status	↔(Aadamsoo *et al*., 2011; Fusar-Poli *et al*., 2017a)
Polymorphic symptomatology	↓(Rusaka and Rancans, 2014a); ↔(Suda *et al*., 2005; Rusaka and Rancans, 2014b)
Poor premorbid social functioning	↑(Suda *et al*., 2005); ↔(Suda *et al*., 2005; Rusaka and Rancans, 2014b)
Quality of life	↔(Aadamsoo *et al*., 2011)
Seriously disorganising or dangerous symptoms	↑(Fusar-Poli *et al*., 2017a, 2019a, 2019b, 2019c)
Severity of psychotic symptoms	↔(Aadamsoo *et al*., 2011; Fusar-Poli *et al*., 2017a)
Sleep disturbances	↑ (Suda *et al*., 2005); ↔(Suda *et al*., 2005; Rusaka and Rancans, 2014a, 2014b)
Thought disorder	↑(Suda *et al*., 2005); ↔(Suda *et al*., 2005; Rusaka and Rancans, 2014a, 2014b)
Years of education	↔(Aadamsoo *et al*., 2011)
Younger age	↑(Abe *et al*., 2006; Aadamsoo *et al*., 2011; Queirazza *et al*., 2014; Poon and Leung, 2017; Wang *et al*., 2018); ↔(Suda *et al*., 2005; Rusaka and Rancans, 2014b; Fusar-Poli *et al*., 2017a)

BPE, brief psychotic episodes.

↑: predictor is associated with higher risk of psychotic recurrence.

↔: predictor is not associated with a change in psychotic recurrence.

↓: predictor is associated with lower risk of psychotic recurrence.

### Sensitivity analyses

Excluding one study at a time at each follow-up point for primary outcome confirmed the robustness of the findings (sensitivity plots are presented in the online Supplementary eFigs 2a–d).

### Heterogeneity and multiple meta-regression

Heterogeneity (*I*^2^) ranged from 43.8% at 24 months to 84.6% at ⩾36 months for risk of psychotic recurrences (online Supplementary eResults 1). Significant multiple meta-regression analyses (online Supplementary eTable 4) associated the female ratio (*β* = 0.0040; *f* = 4.03, *p* = 0.022) with a lower proportion of recurrence; study quality (*β* = 0.0018; *f* = 3.47, *p* = 0.036) and with higher proportion of recurrence (scatterplots are depicted in online Supplementary eFigs 3 and 4).

### Publication bias

Visual inspection of the funnel plot did not reveal publication bias. Egger's test for funnel plot asymmetry was not significant (*p* = 0.27). There was no meta-regression association between sample size and psychotic recurrence (online Supplementary eFigs 5a–c).

## Discussion

To our best knowledge, this is the first systematic review and meta-analysis to have appraised multiple clinical outcomes across several operationalisations of BPE. We showed that these patients have a high risk of developing psychotic recurrences, in particular schizophrenia spectrum psychoses, and high proportion of baseline unemployment, while there were not enough data to explore remission, quality of life and mortality and no consistent factors predicting outcomes. As one in two patients presenting with short-lived psychotic episodes is at risk of recurrences, close monitoring and secondary preventive strategies should be systematically implemented for this vulnerable patient group.

This is the largest meta-analysis of BPE, which encompasses four operationalisations (ATPD, BPD, BLIPS and BIPS) over 91 independent articles and 15 729 individuals. Their mean age of about 31 years falls in the upper range of the 15–35-year window for highest risk of psychosis in the general population (Radua *et al*., 2018). A recent meta-analysis of epidemiological studies completed by our group confirmed that the peak age at onset is 18.5 years, and the median age of onset for ATPD is past 30 years (at 35 years), compared to 25 years for schizophrenia spectrum disorder (Solmi *et al*., 2021). Despite a variable pattern of course and outcome reported by the individual studies, reflected by the high levels of heterogeneity in our analyses, we found that about one-third of patients with BPE will develop a psychotic recurrence after 3 years. In the short term, the BIPS group showed a higher risk of psychotic recurrence. This finding is in line with previous evidence highlighting the substantial higher level of risk in BLIPS/BIPS subgroups compared to other CHR-P subgroups (APS and GRD) (Fusar-Poli *et al*., 2016b). The higher risk of recurrence in BIPS might be related to its 3-month duration criteria, compared to 7-days in BLIPS and 1 month in BPD and most ATPD subtypes. A longer duration of baseline psychotic symptoms is expected to increase the risk of recurrence (Aadamsoo *et al*., 2011; Queirazza *et al*., 2014; Poon and Leung, 2017; Wang *et al*., 2018). We also demonstrated that the presence of seriously disorganising or dangerous features within BLIPS/BIPS is associated with an extreme risk of psychotic recurrence over time (90% at 5-years) (Fusar-Poli *et al*., 2017a). However, these differences of risk for psychotic recurrences across operationalisations were not maintained in the long term. Importantly, we observed that the risk of psychotic recurrence continues increasing up to 3 years, suggesting that these patients should be offered clinical monitoring and relapse prevention strategies for at least 3 years after their index episode (Fusar-Poli *et al*., 2017b, 2020; Rutigliano *et al*., 2018). Unfortunately, first-episode services tend to discharge patients who have spontaneously remitted from an initial episode of psychosis, whereas CHR-P services typically cap their care at 2 years only (the average duration of care in these teams is of 24 months; Salazar de Pablo *et al*., 2021). Consequently, patients with BPE have several unmet needs that are not addressed by current mental health services. For example, only 3% of BLIPS engage with the minimum effective dose of cognitive behavioural therapy, which is also of questionable efficacy for this patient population (Fusar-Poli *et al*., 2019a, *2019b*, *2021a*).

We also showed that about half of patients with BPE will retain the initial diagnoses, compared to 90% in schizophrenia (90%) (Fusar-Poli *et al*., 2016c). About 19% of BPE is being rediagnosed as schizophrenia spectrum psychoses, 5% as affective spectrum psychoses (higher in BPD than ATPD), 7% as other psychotic disorders (higher in BPD than ATPD) and 14% other (non-psychotic) mental disorders. These findings corroborate the conceptual foundations of BPE, which are not static nosological entities (Ey, 1954; Pillmann and Marneros, 2003), but have an intrinsically dynamic nature. Diagnostic changes are therefore to be expected, reflecting the natural trajectory of the disorders. This presents an unprecedented empirical prognostic and preventive opportunity for clinical practice, overcoming the sterile discussion about prospective instability of static diagnostic snapshots (Fusar-Poli *et al*., 2018).

Notably, our sensitivity analyses provided useful data in view of the forthcoming ICD-11 revisions. The APPD without symptoms of schizophrenia showed a 21% meta-analytic risk of psychotic recurrence (at 51 months), a prospective diagnostic stability comparable to the whole ATPD group (56 *v.* 49%), as well as a comparable proportion of diagnostic change to schizophrenia spectrum psychoses (18 *v.* 19%) and of other (non-psychotic) mental disorders (17 *v.* 14%). These findings do not align with the arguments made by the ICD-11 Panel that APPD is ‘not indicative of schizophrenia’ (Reed *et al*., 2019). The ICD-11 field study also showed that the ICD-11 ATPD diagnostic reliability (moderate: kappa = 0.45) is lower than for the ICD-10 ATPD (Reed *et al*., 2018). Some authors have argued that ICD-11 ATPD category is too narrow and may counter-productively reduce its clinical utility (Castagnini and Berrios, 2019).

We also uncovered gaps of knowledge because evidence relating to remission, functional status, quality of life and mortality in brief psychoses is still to sparse and insufficient. For example, most studies addressing quality of life focused on first-episode psychosis without distinguishing across short lived and persistent disorders (Sevilla-Llewellyn-Jones *et al*., 2019; Tan *et al*., 2019; Satghare *et al*., 2020). We were only able to meta-analyse the proportion of patients with brief psychoses who were employed at baseline (48%) and we uncovered no robust and replicated predictors of any outcomes in short-lived psychotic episodes. Our meta-regressions confirm previous findings underlining reduced risk for transition to schizophrenia in females (Queirazza *et al*., 2014; Radua *et al*., 2018; Wang *et al*., 2018). Overall, it is unlikely that single prognostic factors can be robustly associated with this complex and evolving condition. Some multivariable prognostic models that can forecast clinical outcomes in patients with BPE have been developed (Lopez-Diaz *et al*., 2020; Fusar-Poli, 2021). We have recently developed and internally validated (3018 individuals with short-lived psychotic episodes, up to 8 years of follow-up) an individualised clinical prediction model based on Electronic Health Records which adequately predicts the long-term risk of psychotic recurrence in this patient group (prognostic accuracy = 0.7, Damiani *et al*., 2021). This clinical prediction models retained adequate prognostic performance when tested in the context of the ICD-11 changes to this groups and could further be improved by incorporating a dynamic component (Lopez-Diaz *et al*., 2020; Studerus *et al*., 2020) which better captures the evolving nature of this condition.

Overall, based on the findings of this study best practice affords for patients with short-lived psychotic episodes would require repeated clinical monitoring bolstered by complementary relapse prevention strategies (Alvarez-Jimenez *et al*., 2016) for at least 3 years follow-up, to align with the current early intervention recommendations (Chandra *et al*., 2018). At a research level, larger prospective studies with a harmonised set of assessment and outcome measures are needed to further advance precision psychiatry efforts that are essential in this field. Future clinical and research perspective have been fully elaborated in an independent study (Fusar-Poli *et al*., 2021b). The limitations are appended in the online Supplementary eLimitations.

## Conclusions

Short-lived psychotic episodes are associated with a high risk of psychotic recurrences, in particular schizophrenia spectrum disorders. Other clinical outcomes remain relatively underinvestigated. There are no consistent prognostic/predictive factors.

## Supplementary material

For supplementary material accompanying this paper visit http://dx.doi.org/10.1017/S2045796021000548. click here to view supplementary material

## Availability of data and materials

The authors give no permission to share raw data.
