# Supplementary material for: Clinical outcomes in brief psychotic episodes: a systematic review and meta-analysis
Source: Epidemiol Psychiatr Sci. 2021 Nov 4;30:e71. doi: 10.1017/S2045796021000548 (PMC8581951; doi:10.1017/S2045796021000548)
Supplement: Supplementary file 1 [file epssup.zip › S2045796021000548sup001.docx]

**SUPPLEMENTARY MATERIAL**

**eTable 1:** PRISMA statement and checklist…..…..…..…..…..…..…..…..…..…..…..…..…..….…..…..…..…...…..….…..…..…..…..…page 2-3

**eTable 2:** MOOSE checklist.…..…..…..…..…..…..…..…..…..…..…..…..…..…..…..…..…..…..…..…..….…...…..…..…..…..….…..….page 4-5

**eMethods 1 and 2**…………………………………………………………………………………………………….…………………………...page 6

**eTable 3:** Characteristics of the studies included in meta-analyses..…..……...…..…..…..…...…..…..…..…...….…..…..…..…..…..…page 7-15

**eTable 4:** Meta-regressions of moderators of Risk of psychotic recurrence. …..…..…..…...…..…..…..…...….…..……..…..…..…..…page 16

**eFigure 1:** Meta-analytical outcomes for APPD (acute polymorphic psychotic disorder without symptoms of schizophrenia)

subtype of ATPD…………………………………………………………………………………………………….………………….…………..page 17

**eFigure 2a-d**: Sensitivity analyses for primary outcome (psychotic recurrence)……………………………………………………………page 18-21

**eResults 1**…………………………………………………………………………………………………………………………………..………page 22

**eFigure 3:** Meta-regression scatterplot for Female ratio……………………….…..…..…..…...…..…..…..…..….…..……..…..…..…..…page 23

**eFigure 4:** Meta-regression scatterplot for Study quality…………………..….…..…..…..…...…..…..…..…..….…..……..…..…..…..….page 24

**eFigure5a-c**: Publication bias assessment……………………………………………………………………………………………………...page 25-26

**eTable 5**: List of excluded studies (full text screening)……………………………………………………………….………………………..page 27-30

**eLimitations**…………………………………………………………………………………………………………………………….………….page 31

**References**…………………………………………………………………………………………………………………………………………page 32-38

**This supplementary material has been provided by the authors to give readers additional information about their work.**

**eTable 1: PRISMA statement and checklist**

| **Section/topic** | **#** | **Checklist item** | **Page** |
| --- | --- | --- | --- |
| **TITLE** | | |  |
| Title | 1 | Identify the report as a systematic review, meta-analysis, or both. | 1 |
| **ABSTRACT** | | |  |
| Structured summary | 2 | Provide a structured summary including, as applicable: background; objectives; data sources; study eligibility criteria, participants, and interventions; study appraisal and synthesis methods; results; limitations; conclusions and implications of key findings; systematic review registration number. | 2 |
| **INTRODUCTION** | | |  |
| Rationale | 3 | Describe the rationale for the review in the context of what is already known. | 4 |
| Objectives | 4 | Provide an explicit statement of questions being addressed with reference to participants, interventions, comparisons, outcomes, and study design (PICOS). | 5 |
| **METHODS** | | | |
| Protocol and registration | 5 | Indicate if a review protocol exists, if and where it can be accessed (e.g., Web address), and, if available, provide registration information including registration number. | 6 |
| Eligibility criteria | 6 | Specify study characteristics (e.g., PICOS, length of follow-up) and report characteristics (e.g., years considered, language, publication status) used as criteria for eligibility, giving rationale. | 5-6 |
| Information sources | 7 | Describe all information sources (e.g., databases with dates of coverage, contact with study authors to identify additional studies) in the search and date last searched. | 5-6 |
| Search | 8 | Present full electronic search strategy for at least one database, including any limits used, such that it could be repeated. | 5 |
| Study selection | 9 | State the process for selecting studies (i.e., screening, eligibility, included in systematic review, and, if applicable, included in the meta-analysis). | 5 |
| Data collection process | 10 | Describe method of data extraction from reports (e.g., piloted forms, independently, in duplicate) and any processes for obtaining and confirming data from investigators. | 6 |
| Data items | 11 | List and define all variables for which data were sought (e.g., PICOS, funding sources) and any assumptions and simplifications made. | 6 |
| Risk of bias in individual studies | 12 | Describe methods used for assessing risk of bias of individual studies (including specification of whether this was done at the study or outcome level), and how this information is to be used in any data synthesis. | 6-7 |
| Summary measures | 13 | State the principal summary measures. | 7 |
| Risk of bias across studies | 15 | Specify any assessment of risk of bias (i.e. Newcastle-Ottawa Scale (NOS), that may affect the cumulative evidence. | 6 |
| Additional analyses | 16 | Describe methods of additional analyses (e.g., sensitivity or subgroup analyses, meta-regression), if done, indicating which were pre-specified. | 7-8 |
| **RESULTS**   | | | |
| Study selection | 17 | Give numbers of studies screened, assessed for eligibility, and included in the review, with reasons for exclusions at each stage, ideally with a flow diagram. | 8, Figure1 |
| Study characteristics | 18 | For each study, present characteristics for which data were extracted (e.g., study size, PICOS, follow-up period) and provide the citations. | 8, eTable3 |
| Risk of bias within studies | 19 | Present data on risk of bias of each study and, if available, any outcome level assessment (see item 12) | - |
| Results of individual studies | 20 | For all outcomes considered (benefits or harms), present, for each study a summary data for each intervention group | - |
| Synthesis of results | 21 | Present results of study analyzed. | 8-10 |
| Risk of bias across studies | 22 | Present results of any assessment of risk of bias across studies (see Item 15). | 10 |
| Additional analysis | 23 | Give results of additional analyses, if done (e.g., sensitivity or subgroup analyses, meta-regression [see Item 16]). | 10-11 |
| **DISCUSSION**   | | | |
| Summary of evidence | 24 | Summarize the main findings including the strength of evidence for each main outcome; consider their relevance to key groups (e.g., healthcare providers, users, and policy makers). | 11-12 |
| Limitations | 25 | Discuss limitations at study and outcome level (e.g., risk of bias), and at review-level (e.g., incomplete retrieval of identified research, reporting bias). | 13 |
| Conclusions | 26 | Provide a general interpretation of the results in the context of other evidence, and implications for future research. | 13 |
| **FUNDING**   | | | |
| Funding | 27 | Describe sources of funding for the systematic review and other support (e.g., supply of data); role of funders for the systematic review. | - |

**eTable 2: MOOSE Statement - Reporting Checklist for Authors, Editors, and Reviewers of Meta-analyses of Observational Studies**

| **Reporting Criteria** | **Reported (Yes/No)** | **Reported on Page** |
| --- | --- | --- |
| **Reporting of Background** |  |  |
| Problem definition | Yes | 5 |
| Hypothesis statement | Yes | 5 |
| Description of Study Outcome(s) | Yes | 5 |
| Type of exposure or intervention used | Yes | 5 |
| Type of study design used | Yes | 5-6 |
| Study population | Yes | 5-6 |
| **Reporting of Search Strategy** |  |  |
| Qualifications of searchers (eg, librarians and investigators) | Yes | 6 |
| Search strategy, including time period included in the synthesis and keywords | Yes | 5 |
| Effort to include all available studies, including contact with authors | Yes | 6 |
| Databases and registries searched | Yes | 5 |
| Search software used, name and version, including special features used (eg, explosion) | No | - |
| Use of hand searching (eg, reference lists of obtained articles) | Yes | 5 |
| List of citations located and those excluded, including justification |  |  |
| Method for addressing articles published in languages other than English | Yes | 5 |
| Method of handling abstracts and unpublished studies | Yes | 5 |
| Description of any contact with authors | No | - |
| **Reporting of Methods** |  |  |
| Description of relevance or appropriateness of studies assembled for assessing the hypothesis to be tested | Yes | 5-6 |
| Rationale for the selection and coding of data (eg, sound clinical principles or convenience) | Yes | 6 |
| Documentation of how data were classified and coded (eg, multiple raters, blinding, and interrater reliability) | Yes | 6 |
| Assessment of confounding (eg, comparability of cases and controls in studies where appropriate | No | - |
| Assessment of study quality, including blinding of quality assessors; stratification or regression on possible predictors of study results | Yes | 6-7 |
| Assessment of heterogeneity | Yes | 7 |
| Description of statistical methods (eg, complete description of fixed or random effects models, justification of whether the chosen models account for predictors of study results, dose-response models, or cumulative meta-analysis) in sufficient detail to be replicated | Yes | 7-8 |
| Provision of appropriate tables and graphics | Yes | Figure 1-3, Table 1.-3 |
| **Reporting of Results** |  |  |
| Table giving descriptive information for each study included |  | eTable3 |
| Results of sensitivity testing (eg, subgroup analysis) |  | eFigure4 |
| Indication of statistical uncertainty of findings | No | - |
| **Reporting of Discussion** |  |  |
| Quantitative assessment of bias (eg, publication bias) | Yes | 10 |
| Justification for exclusion (eg, exclusion of non–English-language citations) | Yes | Figure 1, eTable 5 |
| Assessment of quality of included studies | No | - |
| **Reporting of Conclusions** |  |  |
| Consideration of alternative explanations for observed results | Yes | 11-12 |
| Generalization of the conclusions (ie, appropriate for the data presented and  within the domain of the literature review) | Yes | 11-12 |
| Guidelines for future research | Yes | 12 |
| Disclosure of funding source | Yes | 13 |

**eMethods 1 and 2**

**eMethods 1: Details of the literature search**

Web of Science database (Clarivate Analytics) was searched, incorporating the Web of Science Core Collection, BIOSIS Citation Index, KCI Korean Journal Database, MEDLINE, Russian Science Citation Index, and SciELO Citation Index. The following search string was used:

((acute and transient psych* OR ATPD OR "brief psych*" OR BPD OR brief intermittent psych* symptoms OR BIPS OR brief limited intermittent psych* symptoms OR BLIPS OR Acute Polymorphic Psych* Disorder OR APPD) AND (first episode psychosis OR transition OR conversion OR relapse OR recurrence OR diagnostic instability OR diagnostic change OR quality of life OR mortality OR predictor* OR functioning OR outcomes)).

**eMethods 2: Variables extracted**

From each study, a predetermined set of variables which were necessary to characterise the study and measure the outcomes or predictors was included: study name and year of publication, country (continent), brief psychotic episode (diagnosis) classification (ATPD/BPD/BIPS/BLIPS), classification instrument (CAARMS/SIPS/DSM/ICD), operationalisation of psychotic recurrence at follow-up, baseline sample size, age (mean ±SD), gender (female ratio), follow-up (months), outcome data and types of predictors.

**eTable 3: Characteristics of the studies included in meta-analyses.**

| **Study name and year of publication** | **Country (Continent)** | **Brief Psychotic Episode (Diagnosis) classification** | **Classification Instrument** | **Operationalisation of psychotic recurrence at follow-up** | **Baseline sample size** | **Age**  **(mean ±SD)** | **Female ratio** | **Follow-up (months)** | **Outcomes** | **Predictors** |
| --- | --- | --- | --- | --- | --- | --- | --- | --- | --- | --- |
| Aadamsoo, et al. 2011 (Aadamsoo *et al.*, 2011) | Estonia (Europe) | ATPD, APPD | ICD-10 | ICD-10 | 153 ATPD, 38 APPD | 27.8±8.2 | 60.1 | 24 | Psychotic recurrence, diagnostic stability, diagnostic change, quality of life | Psychotic recurrence, diagnostic stability |
| Abe, et al. 2006 (Abe *et al.*, 2006) | Japan (Asia) | ATPD (APPD only) | ICD-10 | ICD-10 | 16 | 34.8±9.9 | 50.0 | 144 | Psychotic recurrence, diagnostic stability, diagnostic change | Psychotic recurrence |
| Addington, et al. 2006 (Addington *et al.*, 2006) | Canada (North America) | BPD | DSM-IV | DSM-IV | 5 | 24.5±8.2 | 32.9 | 12 | Psychotic recurrence, diagnostic stability, diagnostic change | n.a. |
| Addington, et al. 2015 (Addington *et al.*, 2015) | United States; Canada (North America) | BIPS | SIPS | SIPS | 20 | 18.4±4.2 | 42.9 | 24 | Psychotic recurrence | n.a. |
| Amini, et al. 2005 (Amini *et al.*, 2005) | Iran (Asia) | ATPD, BPD | ICD-10, DSM-IV | ICD-10, DSM-IV | 10 ATPD, 8 BPE | 24.7±9.5 | 45.0 | 12 | Psychotic recurrence, diagnostic stability, diagnostic change | n.a. |
| Armando, et al. 2015 (Armando *et al.*, 2015) | Italy (Europe) | BIPS | SIPS | SIPS | 5 | 13.8±2.1 | 48.6 | 12 | Psychotic recurrence | n.a. |
| Baldwin, et al. 2005 (Baldwin *et al.*, 2005) | Ireland (Europe) | BPD | DSM-IV | DSM-IV | 13 | 36.5±8.6 | 80.0 | 6 | Psychotic recurrence, diagnostic stability, diagnostic change | n.a. |
| Barak, et al. 2011 (Barak *et al.*, 2011) | Israel (Asia) | ATPD | ICD-10 | ICD-10 | 1 | 75.4±9.3 | 56.6 | 28 | Psychotic recurrence, diagnostic stability, diagnostic change | n.a. |
| Björkenstam, et al. 2013 (Bjorkenstam *et al.*, 2013) | Sweden (Europe) | ATPD | ICD-10 | ICD-10 | 868 | 21.3±3.6 | 65.5 | 60 | Psychotic recurrence, diagnostic stability, diagnostic change, remission | n.a. |
| Caseiro, et al. 2012 (Caseiro *et al.*, 2012) | Spain (Europe) | BPD | DSM-IV | DSM-IV | 13 | 26.3±7.2 | 38.0 | 36 | Psychotic recurrence, diagnostic stability, diagnostic change | n.a. |
| Castagnini and Foldager, 2014 (Castagnini and Foldager, 2014) | Denmark (Europe) | ATPD, APPD | ICD-10 | ICD-10 | 5426 ATPD, 1108 APPD | 28.0±11.7 | 47.4 | 112 | Psychotic recurrence, diagnostic stability, diagnostic change | Diagnostic stability |
| Castagnini et al. 2008 (Castagnini *et al.*, 2008) | Denmark (Europe) | ATPD | ICD-10 | ICD-10 | 503 | 42.2±20.2 | 51.7 | 36 | Psychotic recurrence, diagnostic stability, diagnostic change, remission, mortality | n.a. |
| Castagnini et al, 2016 (Castagnini *et al.*, 2016) | Denmark (Europe) | ATPD, APPD | ICD-10 | ICD-10 | 46 | 36.3±12.5 | 63.8 | 12 | Psychotic recurrence, diagnostic stability, diagnostic change | n.a |
| Castro-Fornieles, et al. 2011 (Castro-Fornieles *et al.*, 2011) | Spain (Europe) | BPD | DSM-IV | DSM-IV | 5 | 15.5±1.7 | 32.5 | 24 | Psychotic recurrence, diagnostic stability, diagnostic change, remission | n.a. |
| Chang, et al. 2009 (Chang *et al.*, 2009) | Hong Kong (Asia) | ATPD, APPD | ICD-10 | ICD-10 | 17 ATPD, 2 APPD | 19.8±3.1 | 46.4 | 53 | Psychotic recurrence, diagnostic stability, diagnostic change | n.a. |
| Chen, et al. 2010 (Chen *et al.*, 2010) | Hong Kong (Asia) | BPD | DSM-IV | DSM-IV | 21 | 24.9±7.3 | 54.0 | 12 | Psychotic recurrence, diagnostic stability, diagnostic change | n.a. |
| Correll, et al. 2005 (Correll *et al.*, 2005) | United States (North America) | BPD | DSM-IV | DSM-IV | 5 | 18.1±3.2 | 20.0 | 25 | Psychotic recurrence, diagnostic stability, diagnostic change | n.a. |
| Das, et al. 1999 (Das *et al.*, 1999) | India (Asia) | ATPD | ICD-10 | ICD-10 | 40 | 25.7±9 | 60.0 | 3 | Psychotic recurrence, diagnostic stability, diagnostic change, functional status | n.a. |
| Dragt et al. 2011 (Dragt *et al.*, 2011) | Netherlands (Europe) | BIPS | SIPS | SIPS | 11 | 19.3±4 | 34.7 | 36 | Psychotic recurrence | n.a. |
| Esan, et al. 2013 (Esan and Fawole, 2013) | Nigeria (Africa) | ATPD | ICD-10 | ICD-10 | 124 | 29.5±9.6 | 50.8 | n.a. | Functional status | Remission |
| Fraguas, et al. 2008 (Fraguas *et al.*, 2008) | Spain (Europe) | BPD | DSM-IV | DSM-IV | 2 | 15.7±1.6 | 25.0 | 24 | Psychotic recurrence, diagnostic stability, diagnostic change | n.a. |
| Fusar-Poli, et al. 2014 (Fusar-Poli *et al.*, 2014) | United Kingdom; Australia (Europe and Australia) | BLIPS | CAARMS | CAARMS | 29 | 18.46±3.44 | 59.0 | 42 | Psychotic recurrence | n.a. |
| Fusar-Poli, et al., 2017 (Fusar-Poli *et al.*, 2017) | United Kingdom (Europe) | BLIPS | CAARMS | CAARMS | 62 | 25.1±5.4 | 41.3 | 60 | Psychotic recurrence, functional status | Psychotic recurrence |
| Fusar-Poli et al., 2019 (Fusar-Poli *et al.*, 2019) | United Kingdom (Europe) | BLIPS | CAARMS | CAARMS | 102 | 24.8±5.8 | 44.1 | 60 | Psychotic recurrence, functional status | Psychotic recurrence |
| Fusar-Poli, et al. 2012 (Fusar-Poli *et al.*, 2012) | Italy (Europe) | BLIPS | CAARMS | DSM-IV | 1 | 20.7±5.3 | 52.5 | 12 | Psychotic recurrence | n.a. |
| Haahr, et al. 2008 (Haahr *et al.*, 2008) | Norway; Denmark (Europe) | BPD | DSM-IV | DSM-IV | 20 | 27.8±9.6 | 41.5 | 24 | Psychotic recurrence, diagnostic stability, diagnostic change | n.a. |
| Heslin, et al 2015 (Heslin *et al.*, 2015) | United Kingdom (Europe) | ATPD, BPD | ICD-10 | ICD-10 | 24 ATPD, 13 BPD | 30.8±10.7 | 42.1 | 129 | Psychotic recurrence, diagnostic stability, diagnostic change | n.a. |
| Hollis, 2000 (Hollis, 2000) | United Kingdom (Europe) | BPD | DSM-III | DSM-III | 7 | 14.4±1.6 | 52.0 | 133 | Psychotic recurrence, diagnostic stability, diagnostic change | n.a. |
| Huguelet, et al. 2001 (Huguelet *et al.*, 2001) | Switzerland (Europe) | BPD | DSM-III | DSM-III | 23 | N.a. | 61.0 | 45 | Psychotic recurrence, diagnostic stability, diagnostic change | n.a. |
| Jäger, et al. 2003 (Jager *et al.*, 2003) | Germany (Europe) | ATPD | ICD-10 | ICD-10 | 94 | 33.1±10.6 | 48.0 | 60 | Psychotic recurrence, diagnostic stability, diagnostic change | n.a. |
| Jørgensen, et al. 1995 (Jørgensen, 1995) | Denmark (Europe) | ATPD | ICD-10 | ICD-10 | 15 | 38.0±10 | 60.0 | 96 | Psychotic recurrence, diagnostic stability, diagnostic change | n.a. |
| Jørgensen, et al. 1997 (Jørgensen *et al.*, 1997) | Denmark (Europe) | ATPD | ICD-10 | ICD-10 | 51 | 37.0±n.a. | 76.5 | 12 | Psychotic recurrence, diagnostic stability, diagnostic change, functional status | n.a. |
| Katsura, et al. 2014 (Katsura *et al.*, 2014) | Japan (Asia) | BLIPS | CAARMS | CAARMS | 4 | 20.0±4.3 | 62.3 | 29 | Psychotic recurrence | n.a. |
| Kéri, et al. 2009 (Kéri *et al.*, 2009) | Hungary (Europe) | BLIPS | CAARMS | DSM-IV | 6 | 21.2±3.6 | 42.3 | 12 | Psychotic recurrence | n.a. |
| Kim, et al. 2011 (Kim *et al.*, 2011) | South Korea (Asia) | BPD | DSM-IV | DSM-IV | 9 | 27.2±9.5 | 61.3 | 27 | Psychotic recurrence, diagnostic stability, diagnostic change | n.a. |
| Kim, et al. 2012 (Kim *et al.*, 2012) | South Korea (Asia) | BLIPS | CAARMS | DSM-IV | 1 | 21.3±4.2 | 32.0 | 60 | Psychotic recurrence | n.a. |
| Kingston, et al. 2013 (Kingston *et al.*, 2013) | Ireland (Europe) | BPD | DSM-IV | DSM-IV | 13 | 36.5±8.6 | 80.0 | 77 | Psychotic recurrence, diagnostic stability, diagnostic change | n.a. |
| Koike, et al. 2013 (Koike *et al.*, 2013) | Japan (Asia) | BIPS | SIPS | DSM-IV | 3 | 21.3±3.6 | 45.9 | 24 | Psychotic recurrence | n.a. |
| Kotlicka-Antczak, et al. 2014 (Kotlicka-Antczak *et al.*, 2015) | Poland (Europe) | BLIPS | CAARMS | ICD-10 | 4 | 19.1±3.6 | 51.1 | 38 | Psychotic recurrence | n.a. |
| Koutsouleris et al. 2009 (Koutsouleris *et al.*, 2009) | Germany (Europe) | BLIPS | CAARMS | ICD-10 | 17 | 25.1±5.8 | 37.8 | 48 | Psychotic recurrence | n.a. |
| Kumar Kar, et al. 2017 (Kar and Dhanasekaran, 2017) | India (Asia) | ATPD, APPD | ICD-10 | ICD-10 | 140 ATPD, 22 APPD | 26.3±3.5 | 57.9 | 3 | Psychotic recurrence, diagnostic stability, diagnostic change | n.a. |
| Labad, et al. 2015 (Labad *et al.*, 2015) | Spain (Europe) | BLIPS | CAARMS | CAARMS | 7 | 22.3±4.6 | 30.8 | 12 | Psychotic recurrence, | n.a. |
| Lam, et al. 2006 (Lam *et al.*, 2006) | Hong Kong (Asia) | BLIPS | CAARMS | DSM-IV | 12 | 16.2±3.7 | 41.9 | 24 | Psychotic recurrence | n.a. |
| Langbein et al., 2017 (Langbein *et al.*, 2018) | Germany (Europe) | BLIPS | CAARMS | DSM-IV | 13 | 20.7±5 | 8.3 | 12 | Psychotic recurrence | n.a. |
| Lee, et al. 2013 (Lee *et al.*, 2013) | Singapore (Asia) | BLIPS | CAARMS | DSM-IV | 6 | 21.3±3.5 | 32.4 | 30 | Psychotic recurrence | n.a. |
| Lee, et al. 2014 (Lee *et al.*, 2014) | South Korea (Asia) | BIPS | CAARMS | DSM-IV | 1 | 21.3±4.2 | 32.0 | 24 | Psychotic recurrence | n.a. |
| Lemos-Giráldez, et al. 2009 (Lemos-Giraldez *et al.*, 2009) | Spain (Europe) | BIPS | SIPS | DSM-IV | 3 | 21.7±3.83 | 34.4 | 36 | Psychotic recurrence | n.a. |
| Lopez-Diaz, 2018 (Lopez-Diaz *et al.*, 2018) | Spain (Europe) | ATPD | ICD-10 | ICD-10 | 44 | 33.3±10.9 | 58.9 | 3 | Psychotic recurrence, diagnostic stability, diagnostic change, functional status | n.a. |
| Lopez-Diaz, 2019 (Lopez-Diaz *et al.*, 2019) | Spain (Europe) | ATPD | ICD-10 | ICD-10 | 76 | 34.2±11 | 60.3 | 24 | Psychotic recurrence, diagnostic stability, diagnostic change, functional status | n.a. |
| Lyne et al. 2012 (Lyne *et al.*, 2012) | Ireland (Europe) | BPD | DSM-IV | DSM-IV | 24 | 31.4±11.1 | n.a. | n.a | Functional status | n.a. |
| Mason, et al. 2004 (Mason *et al.*, 2004) | United Kingdom (Europe) | BLIPS | CAARMS | DSM-IV | 23 | 17.3±2.8 | 47.3 | 26 | Psychotic recurrence | n.a. |
| Matsumoto, et al. 2018 (Matsumoto *et al.*, 2019) | Japan (Asia) | BLIPS | CAARMS, SIPS | DSM-IV | 34 | 21.4±5.5 | 61.5 | 12 | Psychotic recurrence | n.a. |
| Metzler, et al. 2014 (Metzler *et al.*, 2014) | Switzerland (Europe) | BIPS | SIPS | ICD-10 | 6 | 20.5±5.9 | 56.9 | 12 | Psychotic recurrence | n.a. |
| Möller, et al. 2011 (Moller *et al.*, 2011) | Germany (Europe) | ATPD, BPD | ICD-10, DSM-IV | ICD-10, DSM-IV | 54 ATPD, 12 BPD | 35.0±13.1 | 72.0 | 180 | Psychotic recurrence, diagnostic stability, diagnostic change | n.a. |
| Narayanaswamy, et al. 2012 (Narayanaswamy *et al.*, 2012) | India (Asia) | ATPD | ICD-10 | ICD-10 | 57 | 30.7±11.8 | 65.0 | 24 | Psychotic recurrence, diagnostic stability, diagnostic change | n.a. |
| Nelson, et al. 2011 (Nelson *et al.*, 2011) | Australia (Australia) | BLIPS | CAARMS | CAARMS | 36 | 18.0±n.a. | 59.0 | 6 | Psychotic recurrence | n.a. |
| Nieman, et al. 2013 (Nieman *et al.*, 2013) | Germany; Finland; Netherlands; United Kingdom (Europe) | BIPS | SIPS | DSM-IV | 28 | 22.5±5.23 | 44.0 | 18 | Psychotic recurrence | n.a. |
| Okasha, et al. 1993 (Okasha *et al.*, 1993) | Egypt (Asia) | ATPD | ICD-10 | ICD-10 | 63 | 27.0±9.75 | 50.0 | 12 | Psychotic recurrence, diagnostic stability, diagnostic change | n.a. |
| Pedrós, et al. 2009 (Pedros *et al.*, 2009) | Spain (Europe) | BPD | DSM-IV | DSM-IV | 14 | 28.1±8.7 | N.a. | 24 | Psychotic recurrence, diagnostic stability, diagnostic change | n.a. |
| Peralta, et al. 2019 (Peralta *et al.*, 2019) | Switzerland (Europe) | BLIPS | CAARMS | CAARMS | 15 | 25.1±6.9 | 31.0 | 36 | Psychotic recurrence | n.a. |
| Pillmann, et al. 2012 (Pillmann *et al.*, 2012) | Germany (Europe) | ATPD | ICD-10 | ICD-10 | 38 | 35.9±11.2 | 78.0 | 149 | Psychotic recurrence, diagnostic stability, diagnostic change, functional status | n.a. |
| Poon, et al. 2017 (Poon and Leung, 2017) | Hong Kong (Asia) | ATPD | ICD-10 | ICD-10 | 102 | 31.5±11.1 | 86.2 | 240 | Psychotic recurrence, diagnostic stability, diagnostic change | Psychotic recurrence, diagnostic stability |
| Queirazza, et al. 2014 (Queirazza *et al.*, 2014) | United Kingdom (Europe) | ATPD | ICD-10 | ICD-10 | 2923 | 37.4±17.7 | 45.5 | 48 | Psychotic recurrence, diagnostic stability, diagnostic change | Psychotic recurrence, diagnostic stability |
| Rahm, et al. 2007 (Rahm and Cullberg, 2007) | Sweden (Europe) | BPD | DSM-IV | DSM-IV | 21 | N.a. | N.a. | 36 | Psychotic recurrence, diagnostic stability, diagnostic change, remission | n.a. |
| Rajkujmar, et al. 2015 (Rajkumar, 2015) | India (Asia) | ATPD | ICD-10 | ICD-10 | 20 | 36.4±10.9 | 60 | n.a. | Functional status | n.a. |
| Ranjan, et al. 2014 (Ranjan *et al.*, 2014) | Nepal (Asia) | ATPD | ICD-10 | ICD-10 | 30 | N.a. | N.a. | 3 | Psychotic recurrence, diagnostic stability, diagnostic change | n.a. |
| Remberk, et al. 2014 (Remberk *et al.*, 2014) | Poland (Europe) | ATPD | ICD-10 | ICD-10 | 46 | 16.6±1.2 | 50.6 | 96 | Psychotic recurrence, diagnostic stability, diagnostic change | n.a. |
| Riecher-Rössler, et al. 2009 (Riecher-Rossler *et al.*, 2009) | Switzerland (Europe) | BLIPS | CAARMS | CAARMS | 5 | 26.3±8.3 | 39.6 | 65 | Psychotic recurrence | n.a. |
| Rufino, et al. 2005 (Rufino *et al.*, 2005) | Brazil (South America) | BPD | DSM-IV | DSM-IV | 31 | N.a. | N.a. | 19 | Psychotic recurrence, diagnostic stability, diagnostic change | n.a. |
| Rusaka and Rancans, et al. 2014a (Rusaka and Rancans, 2014a) | Latvia (Europe) | ATPD | ICD-10 | ICD-10 | 102 | 35.8±13.4 | 60.7 | 27 | Psychotic recurrence, diagnostic stability, diagnostic change | Psychotic recurrence, diagnostic stability |
| Rusaka and Rancans, et al. 2014b (Rusaka and Rancans, 2014b) | Latvia (Europe) | ATPD | ICD-10 | ICD-10 | 294 | 33.1±12 | 54.0 | 67 | Psychotic recurrence, diagnostic stability, diagnostic change, functional status | Psychotic recurrence, diagnostic stability |
| Rutigliano, et al. 2018 (Rutigliano *et al.*, 2018) | United Kingdom (Europe) | ATPD | ICD-10 | ICD-10 | 3074 | 33.8±14 | 46.9 | 96 | Psychotic recurrence, diagnostic stability, diagnostic change | n.a. |
| Sajith, et al. 2002 (Sajith *et al.*, 2002) | India (Asia) | ATPD (APPD only) | ICD-10 | ICD-10 | 45 | 26.9±10.9 | 71.1 | 37 | Psychotic recurrence, diagnostic stability, diagnostic change | Diagnostic stability |
| Salem, et al. 2009 (Salem *et al.*, 2009) | Arab Emirates (Asia) | ATPD | ICD-10 | ICD-10 | 69 | 27.5±6.6 | 32.4 | 72 | Psychotic recurrence, diagnostic stability, diagnostic change | n.a. |
| Salvatore, et al. 2009 (Salvatore *et al.*, 2009) (Salvatore *et al.*, 2011) | United States (North America) | ATPD, APPD, BPD | ICD-10, DSM | ICD-10, DSM | ATPD 55, APPD 21, BPD 36 | 31.7±13.7 | 45.0 | 24 | Psychotic recurrence, diagnostic stability, diagnostic change | Diagnostic stability |
| Schimmelman, et al. 2005 (Schimmelmann *et al.*, 2005) | Australia (Australia) | BPD | DSM-IV | DSM-IV | 11 | 22.0±3.6 | N.a. | 18 | Psychotic recurrence, diagnostic stability, diagnostic change | n.a. |
| Schultze-Lutter, et al. 2014 (Schultze-Lutter *et al.*, 2014) | Germany (Europe) | BIPS | SIPS | DSM-IV | 45 | 24.9±6 | 37.0 | 48 | Psychotic recurrence | n.a. |
| Schwartz, et al. 2000 (Schwartz *et al.*, 2000) | United States (North America) | BPD | DSM-IV | DSM-IV | 11 | 28.0±n.a. | N.a. | 18 | Psychotic recurrence, diagnostic stability, diagnostic change | n.a. |
| Simon, et al. 2012 (Simon *et al.*, 2012) | Switzerland (Europe) | BIPS | SIPS | DSM-IV | 3 | 20.4±5.2 | 39.7 | 24 | Psychotic recurrence |  |
| Singh, et al. 2004 (Singh *et al.*, 2004) | United Kingdom (Europe) | ATPD | ICD-10 | ICD-10 | 32 | N.a. | 34.4 | 36 | Psychotic recurrence, diagnostic stability, diagnostic change, functional status | Diagnostic stability |
| Spada, et al. 2015 (Spada *et al.*, 2016) | Italy (Europe) | BLIPS | CAARMS | CAARMS | 1 | 16.1±1.0 | 45.5 | 7 | Psychotic recurrence | n.a. |
| Subramaniam, et al. 2007 (Subramaniam *et al.*, 2007) | Singapore (Asia) | BPD | DSM-IV | DSM-IV | 13 | 28.4±6.6 | 49.0 | 24 | Psychotic recurrence, diagnostic stability, diagnostic change | n.a. |
| Suda, et al. 2005 (Suda *et al.*, 2005) | Japan (Asia) | ATPD | ICD-10 | ICD-10 | 25 | 37.8±9.5 | 76.0 | 116 | Psychotic recurrence, diagnostic stability, diagnostic change | Psychotic recurrence |
| Thangadurai, et al. 2006 (Thangadurai *et al.*, 2006) | India (Asia) | ATPD | ICD-10 | ICD-10 | 87 | 29.8±11 | 48.0 | 13 | Psychotic recurrence, diagnostic stability, diagnostic change | n.a. |
| Van Tricht et al. 2010 (van Tricht *et al.*, 2010) | Netherlands (Europe) | BIPS | SIPS | SIPS | 5 | 19.6±3.8 | 31.1 | 36 | Psychotic recurrence | n.a. |
| Veen, et al. 2004 (Veen *et al.*, 2004) | Netherlands (Europe) | BPD | DSM-IV | DSM-IV | 15 | 29.4±9.4 | 30.4 | 30 | Psychotic recurrence, diagnostic stability, diagnostic change | n.a. |
| Wang, et al. 2017 (Wang *et al.*, 2018) | China (Asia) | ATPD | ICD-10 | ICD-10 | 92 | 28.2±9.1 | 62.0 | 39 | Psychotic recurrence, diagnostic stability, diagnostic change | Psychotic recurrence, diagnostic stability |
| Woods, et al. 2009 (Woods *et al.*, 2009) | United States; Canada (North America) | BIPS | SIPS | DSM-IV | 14 | 17.9±4.5 | 40.9 | 36 | Psychotic recurrence | n.a. |
| Zhang, et al. 2014 (Zhang *et al.*, 2014) | China (Asia) | BIPS | SIPS | DSM-IV | 3 | 25.9±7.5 | 49.4 | 24 | Psychotic recurrence | n.a. |
| Ziermans, et al. 2011 (Ziermans *et al.*, 2011) | Netherlands (Europe) | BIPS | SIPS | DSM-IV | 3 | 15.3±1.9 | 38.9 | 24 | Psychotic recurrence | n.a. |

ATPD: acute and transient psychotic disorder; APPD: Acute Polymorphic Psychotic Disorder; BIPS: brief intermittent psychotic symptoms; BLIPS: brief limited intermittent psychotic symptoms; BPD: brief psychotic disorder; CAARMS: Comprehensive Assessment of At-Risk Mental States; DSM: Diagnostic and Statistical Manual of Mental Disorders ; ICD: International Classification of Diseases; SIPS: Structured Interview for Prodromal Symptoms.

**eTable4. Meta-regressions of Moderators of Risk of psychotic recurrence**

| **Covariate** | **n** | **β** | **Intercept** | **t** | **f** | **p** | **95% CI** |  | **R^2^** |
| --- | --- | --- | --- | --- | --- | --- | --- | --- | --- |
|  |  |  |  |  |  |  |  |  |  |
| Age (y) | 76 | -0,0015 | -0,9635 | -0,52 |  | 0,6027 | -0,0072 | 0,0042 |  |
| Follow up (mo) | 76 | 0,0026 | -0,9635 | 1,33 |  | 0,1887 | -0,0013 | 0,0065 |  |
| Model 1 (mean age + follow up) | 76 |  |  |  | 0.91 | 0.4085 |  |  | 0.00 |
|  |  |  |  |  |  |  |  |  |  |
| Gender (female ratio) | 76 | -0,0176 | -0,1479 | -2,52 |  | 0,0138 | 0,0315 | 0,0037 |  |
| Follow up (mo) | 76 | 0,0040 | -0,1479 | 2,04 |  | 0,0448 | 0,0001 | 0,0078 |  |
| Model 2 (female ratio + follow up) | 76 |  |  |  | 4.03 | *0.0220 |  |  | 0.00 |
|  |  |  |  |  |  |  |  |  |  |
| Publication year (y) | 76 | 0,0180 | -37,1811 | 1,05 |  | 0,2985 | -0,0163 | 0,0523 |  |
| Follow up (mo) | 76 | 0,0021 | -37,1811 | 1,08 |  | 0,2822 | -0,0018 | 0,006 |  |
| Model 3 (publication year + follow up) | 76 |  |  |  | 1.34 | 0.2690 |  |  | 0.00 |
|  |  |  |  |  |  |  |  |  |  |
| Newcastle Ottawa Scale (score) | 76 | 0,1564 | -1,8849 | 2,31 |  | 0,0236 | 0,0216 | 0,2911 |  |
| Follow up (mo) | 76 | 0,0018 | -1,8849 | 0,97 |  | 0,3333 | -0,0019 | 0,0054 |  |
| Model 4 (study quality + follow up) | 76 |  |  |  | 3.47 | *0.0363 |  |  | 0.24 |

**eFigure1. Meta-analytical outcomes for APPD (acute polymorphic psychotic disorder) without symptoms of schizophrenia subtype of ATPD**

**eFigure 2a-d**: Sensitivity analyses for primary outcome (psychotic recurrence)

*eFigure 2a: Sensitivity analyses for primary outcome (psychotic recurrence) at 6 months follow up*


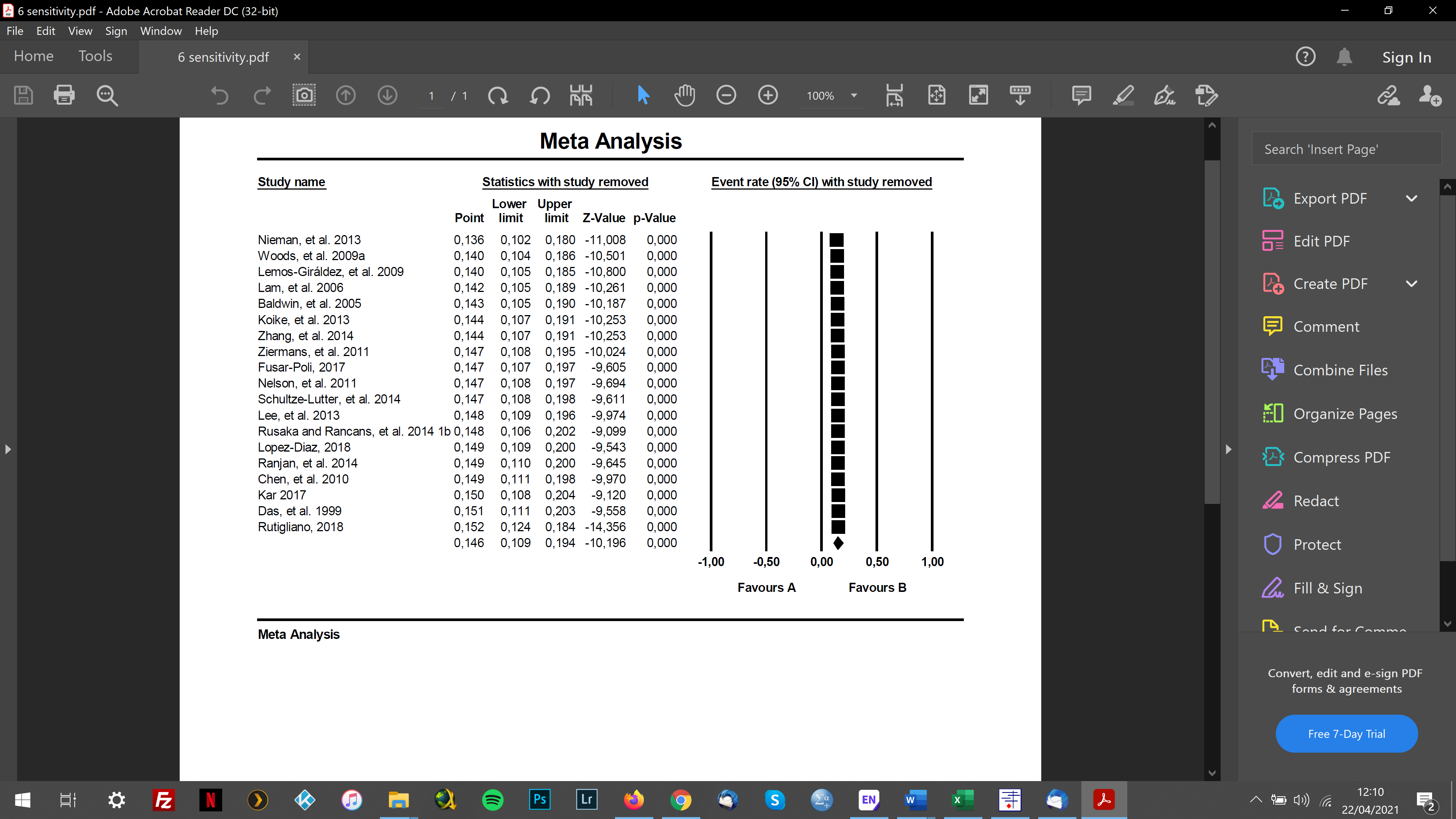


*eFigure 2b: Sensitivity analyses for primary outcome (psychotic recurrence) at 12 months follow up*


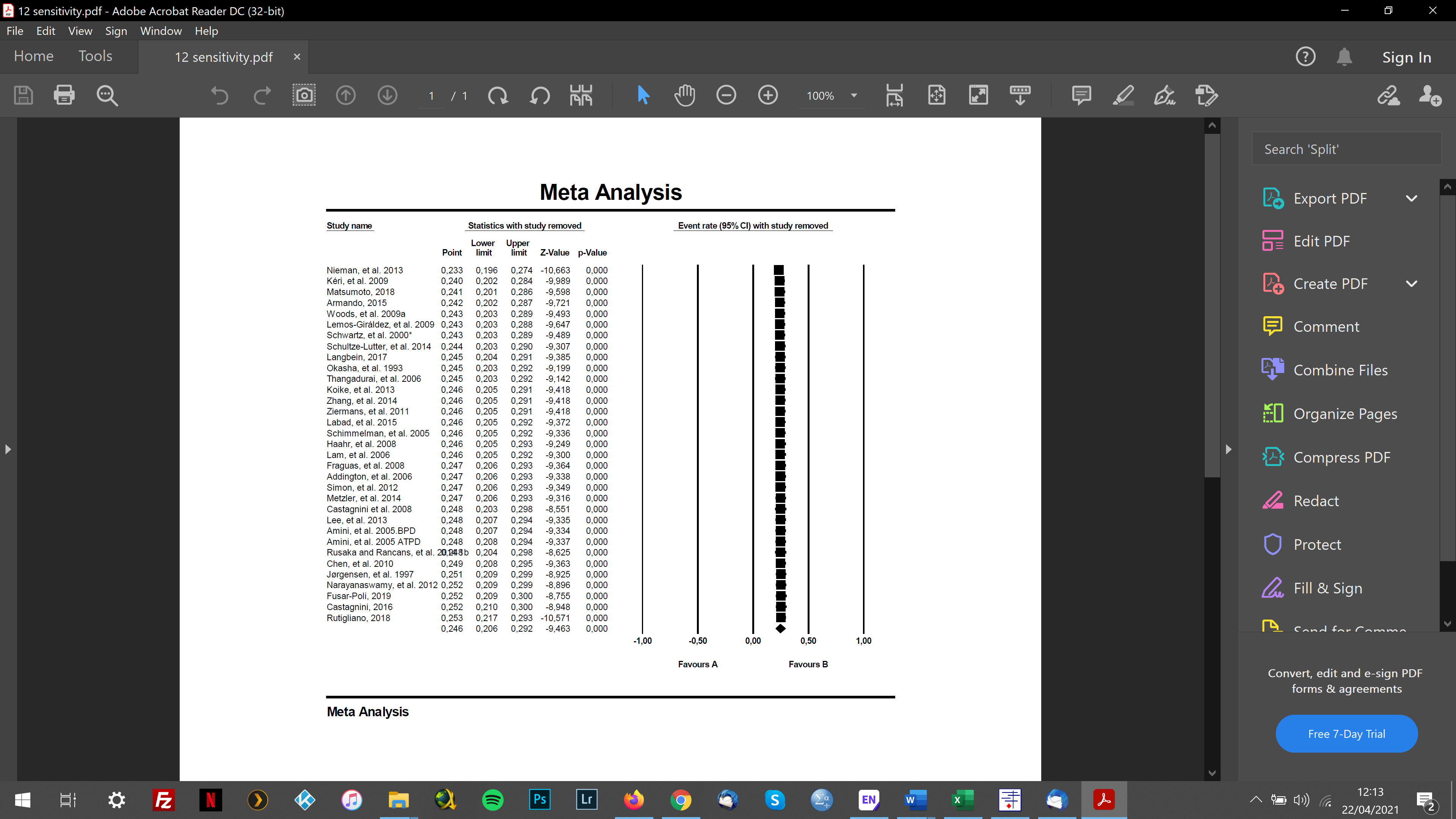


*eFigure 2c: Sensitivity analyses for primary outcome (psychotic recurrence) at 24 months follow up*


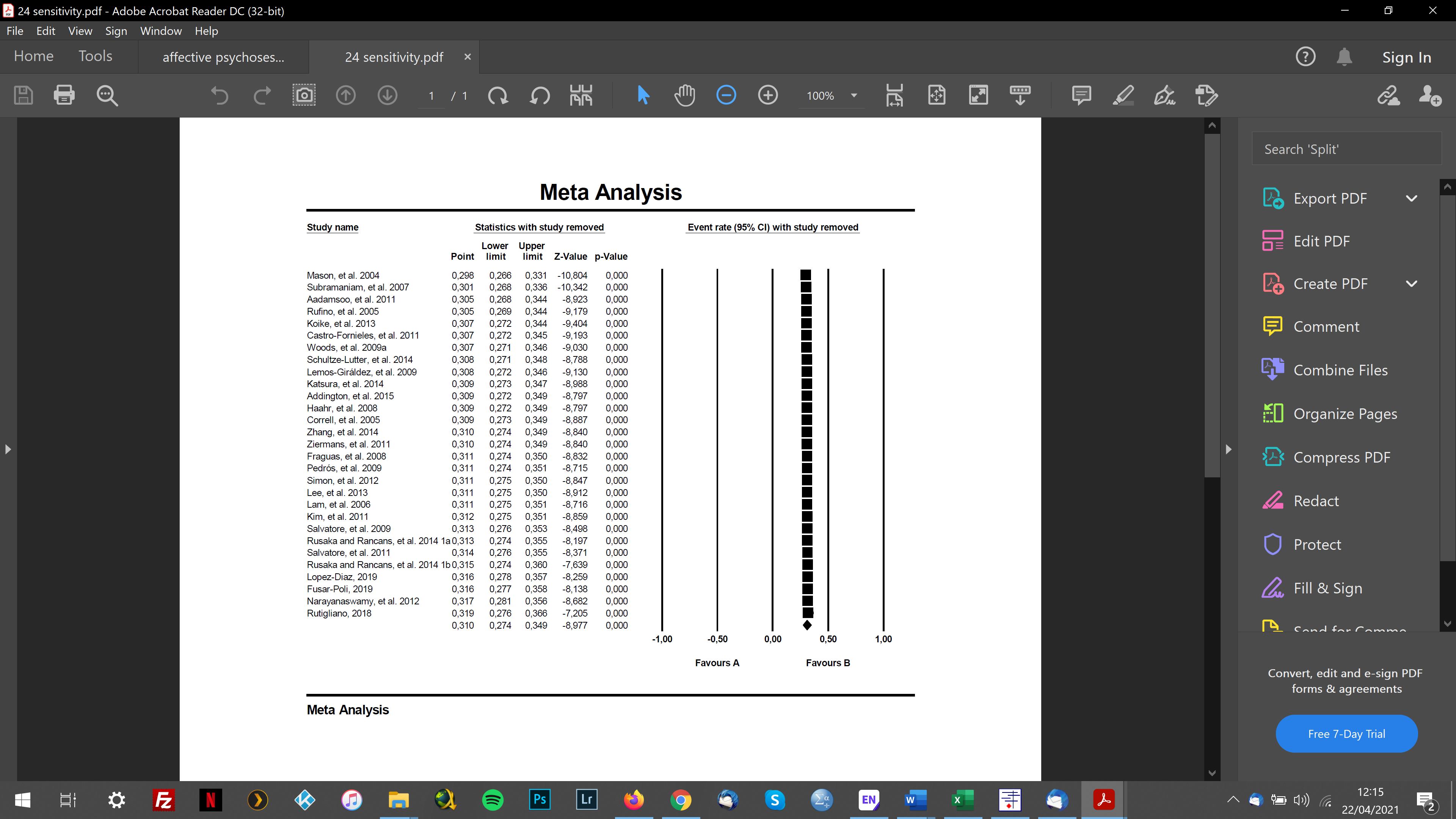


*eFigure 2d: Sensitivity analyses for primary outcome (psychotic recurrence) at ≥36 months follow up*


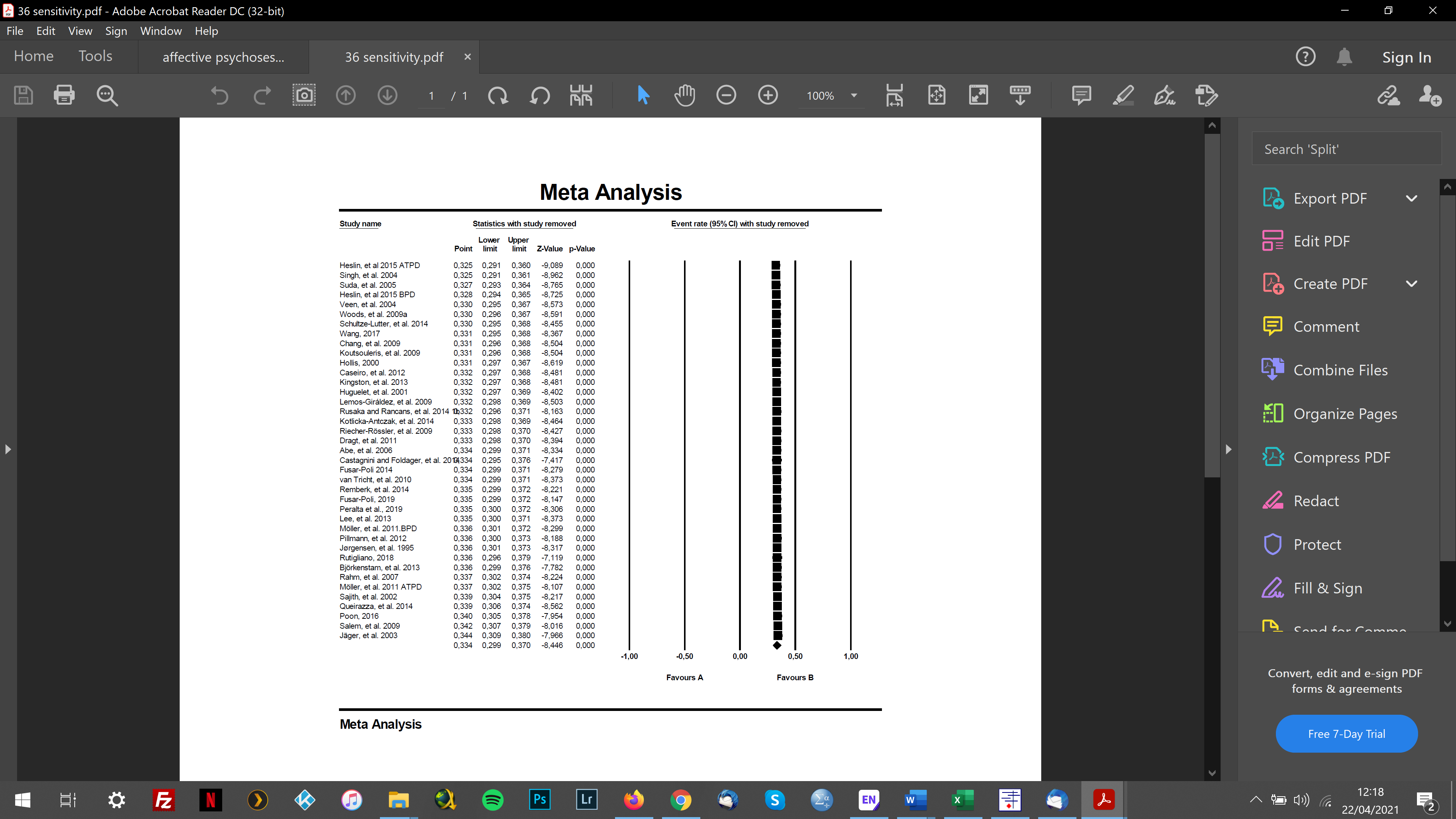


**eResults 1**

Heterogeneity was 97.3% and 97.2% for prospective diagnostic stability and change respectively. Heterogeneity of prospective diagnostic change across specific disorders ranged from 75.6% for affective spectrum psychoses to 94.5% for schizophrenia spectrum psychoses. Heterogeneity of functional/vocational status (vocational outcome) was 86.8%.

**eFigure3. Meta-regression scatterplot for Female ratio**

**Regression of Logit event rate on Female ratio**

**Female ratio**

**0**

**10**

**20**

**30**

**40**

**50**

**60**

**70**

**80**

**90**

**100**

**Logit event rate**

**5,00**

**4,00**

**3,00**

**2,00**

**1,00**

**0,00**

**-1,00**

**-2,00**

**-3,00**

**-4,00**

**-5,00**

**-6,00**

**eFigure4. Meta-regression scatterplot for Study quality**

**Regression of Logit event rate on Study quality**

**Study quality**

**1,0**

**2,0**

**3,0**

**4,0**

**5,0**

**6,0**

**7,0**

**8,0**

**9,0**

**Logit event rate**

**5,00**

**4,00**

**3,00**

**2,00**

**1,00**

**0,00**

**-1,00**

**-2,00**

**-3,00**

**-4,00**

**-5,00**

**-6,00**

**eFigure 5a-c: Publication bias assessment**

*eFigure 5a: Funnel plot of included studies*


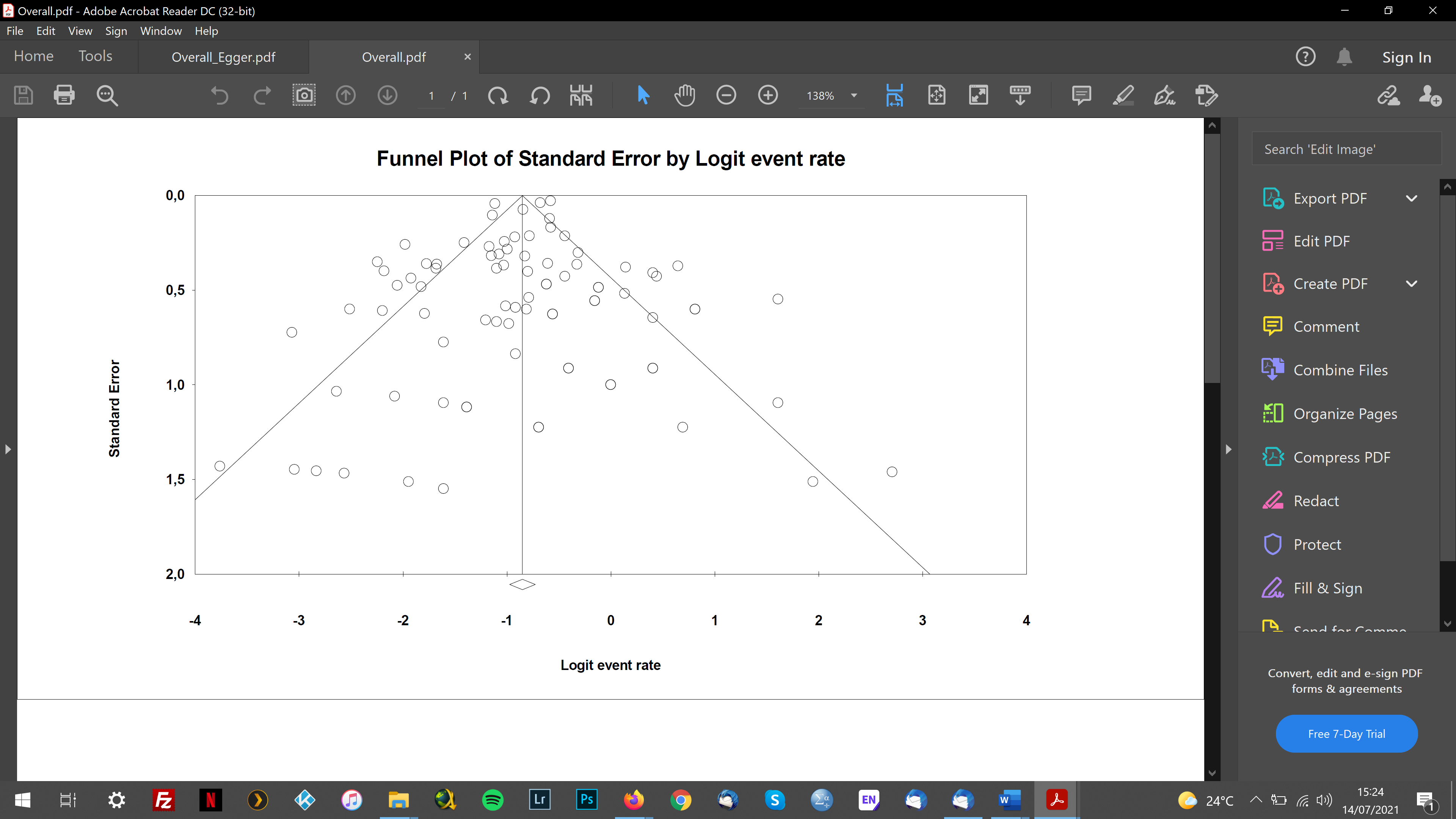


*eFigure 5b: Egger’s Test for funnel plot asymmetry*


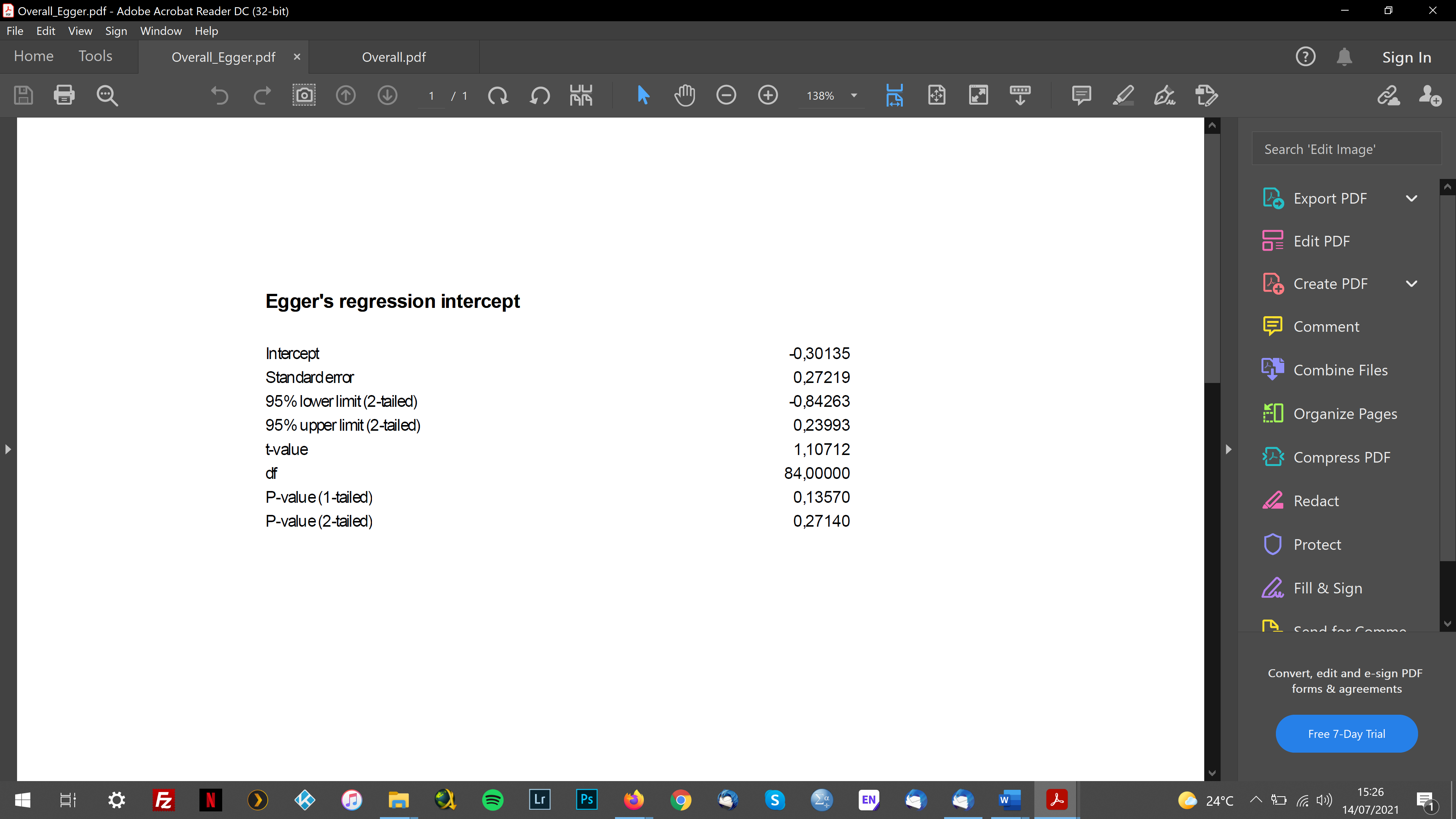


*eFigure 5c: Meta-regression between the main outcome (psychotic recurrence) as dependent variable and sample size as independent variable*


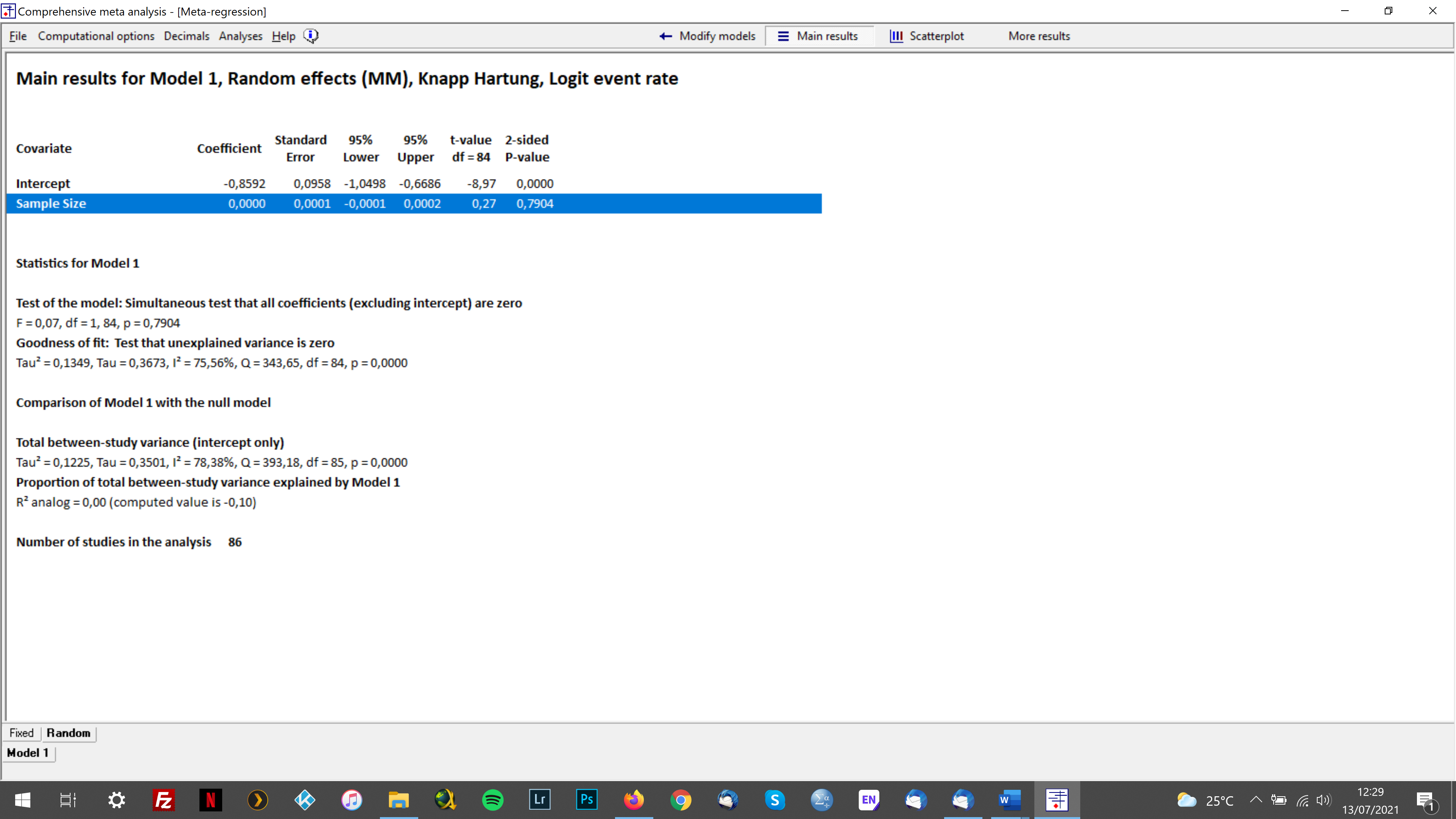


**eTable 5: List of excluded studies (full text screening)**

| **Author** | **Year** | **Endnote Reference** | **Reason for Exclusion** |
| --- | --- | --- | --- |
| Amminger, G. P., et al. | 2011 | {#147} | Exclusion criteria (d) |
| Ayer, A., et al. | 2016 | {#83} | Exclusion criteria (e) |
| Ayesa-Arriola, R., et al. | 2016 | {#148} | Exclusion criteria (d) |
| Basu, S., et al. | 2020 | {#273} | Exclusion criteria (d) |
| Beauchamp, M. C., et al. | 2011 | {#149} | Exclusion criteria (d) |
| Bunevicius, R., et al. | 2014 | {#150} | Exclusion criteria (d) |
| Burley, K., et al. | 2009 | {#151} | Exclusion criteria (d) |
| Castagnini, A. C., et al. | 2011 | {#266} | Exclusion criteria (d) |
| Castagnini, A., et al. | 2011 | {#144} | Exclusion criteria (a) |
| Castagnini, A., et al. | 2013 | {#265} | Exclusion criteria (d) |
| Castagnini, A., et al. | 2013 | {#236} | Exclusion criteria (d) |
| Catalan, A., et al. | 2017 | {#84} | Exclusion criteria (e) |
| Cetty, L., et al. | 2019 | {#272} | Exclusion criteria (d) |
| Chand, P., et al. | 2014 | {#275} | Exclusion criteria (d) |
| Cheli, S., et al. | 2020 | {#152} | Exclusion criteria (d) |
| Chudleigh, C., et al. | 2011 | {#153} | Exclusion criteria (d) |
| Cocchi, A., et al. | 2008 | {#154} | Exclusion criteria (d) |
| Cocchi, L., et al. | 2009 | {#224} | Exclusion criteria (d) |
| Collins, P. Y., et al. | 1996 | {#155} | Exclusion criteria (d) |
| Collins, P. Y., et al. | 1999 | {#85} | Exclusion criteria (e) |
| Comparelli, A., et al. | 2013 | {#156} | Exclusion criteria (d) |
| Comparelli, A., et al. | 2013 | {#157} | Exclusion criteria (d) |
| Cremniter, D., et al. | 2001 | {#86} | Exclusion criteria (e) |
| Cross, S. P., et al. | 2018 | {#87} | Exclusion criteria (e) |
| Dazzi, F., et al. | 2017 | {#88} | Exclusion criteria (e) |
| De Ronchi, D., et al. | 2006 | {#89} | Exclusion criteria (e) |
| Del Bello, V., et al. | 2016 | {#158} | Exclusion criteria (d) |
| Dwyer, D. B., et al. | 2020 | {#159} | Exclusion criteria (d) |
| Edwards, J., et al. | 1998 | {#225} | Exclusion criteria (d) |
| Ehlis, A. C., et al. | 2005 | {#160} | Exclusion criteria (d) |
| Eisenacher, S., et al. | 2018 | {#161} | Exclusion criteria (d) |
| Erdem, M., et al. | 2012 | {#162} | Exclusion criteria (d) |
| Ergul, C., et al. | 2015 | {#90} | Exclusion criteria (e) |
| Esan, O., et al. | 2014 | {#258} | Exclusion criteria (d) |
| Filatova, S., et al. | 2017 | {#163} | Exclusion criteria (d) |
| Flyckt, L., et al. | 2006 | {#164} | Exclusion criteria (d) |
| Frommann, I., et al. | 2011 | {#165} | Exclusion criteria (d) |
| Fulford, D., et al. | 2014 | {#166} | Exclusion criteria (d) |
| Fusar-Poli, P., et al. | 2016 | {#145} | Exclusion criteria (a) |
| Fusar-Poli, P., et al. | 2013 | {#237} | Exclusion criteria (d) |
| Fusar-Poli, P., et al. | 2017 | {#239} | Exclusion criteria (d) |
| Fusar-Poli, P., et al. | 2017 | {#240} | Exclusion criteria (d) |
| Fusar-Poli, P., et al. | 2020 | {#241} | Exclusion criteria (d) |
| Garip, B., et al. | 2019 | {#167} | Exclusion criteria (d) |
| Garner, B., et al. | 2009 | {#168} | Exclusion criteria (d) |
| Gleeson, J. F., et al. | 2005 | {#226} | Exclusion criteria (d) |
| Glenthoj, L. B., et al. | 2015 | {#170} | Exclusion criteria (d) |
| Gonzalez-Blanch, C., et al. | 2010 | {#227} | Exclusion criteria (d) |
| Gonzalez-Rodriguez, A., et al. | 2014 | {#172} | Exclusion criteria (d) |
| Guma, E., et al. | 2017 | {#173} | Exclusion criteria (d) |
| Guzman-Parra, J., et al. | 2018 | {#91} | Exclusion criteria (e) |
| Harrisberger, F., et al. | 2018 | {#174} | Exclusion criteria (d) |
| Helle, S., et al. | 2014 | {#175} | Exclusion criteria (d) |
| Hengartner, M. P., et al. | 2017 | {#176} | Exclusion criteria (d) |
| Hengartner, M. P., et al. | 2017 | {#177} | Exclusion criteria (d) |
| Henry, L. P., et al. | 2010 | {#178} | Exclusion criteria (d) |
| Herniman, S. E., et al. | 2017 | {#228} | Exclusion criteria (d) |
| Hides, L., et al. | 2009 | {#92} | Exclusion criteria (e) |
| Huber, C. G., et al. | 2014 | {#180} | Exclusion criteria (d) |
| Huber, C. G., et al. | 2018 | {#181} | Exclusion criteria (d) |
| Hur, J. W., et al. | 2015 | {#229} | Exclusion criteria (d) |
| Iwawaki, A., et al. | 1996 | {#242} | Exclusion criteria (d) |
| Iyer, S. N., et al. | 2009 | {#93} | Exclusion criteria (e) |
| Jackson, H., et al. | 1998 | {#94} | Exclusion criteria (e) |
| Jager, M., et al. | 2003 | {#243} | Exclusion criteria (d) |
| Johnson, S., et al. | 2014 | {#95} | Exclusion criteria (e) |
| Kalla, O., et al. | 2011 | {#183} | Exclusion criteria (d) |
| Kampman, O., et al. | 2004 | {#184} | Exclusion criteria (d) |
| Kane, J. M., et al. | 2015 | {#185} | Exclusion criteria (d) |
| Karpov, B., et al. | 2015 | {#186} | Exclusion criteria (d) |
| Katz, G., et al. | 2016 | {#187} | Exclusion criteria (d) |
| Kindler, J., et al. | 2016 | {#96} | Exclusion criteria (e) |
| Kirschner, M., et al. | 2018 | {#188} | Exclusion criteria (d) |
| Korner, A., et al. | 2009 | {#271} | Exclusion criteria (d) |
| Korver, N., et al. | 2010 | {#97} | Exclusion criteria (e) |
| Krakvik, B., et al. | 2013 | {#98} | Exclusion criteria (e) |
| Krstev, H., et al. | 1999 | {#99} | Exclusion criteria (e) |
| Kuhl, J. O. G., et al. | 2016 | {#189} | Exclusion criteria (d) |
| Kumar, A., et al. | 2014 | {#190} | Exclusion criteria (d) |
| Lappin, J. M., et al. | 2018 | {#259} | Exclusion criteria (d) |
| Leijala, J., et al. | 2021 | {#100} | Exclusion criteria (e) |
| Lewis, S., et al. | 2002 | {#191} | Exclusion criteria (d) |
| Lho, S. K., et al. | 2019 | {#101} | Exclusion criteria (e) |
| Li, H., et al. | 2020 | {#276} | Exclusion criteria (d) |
| Lin, A., et al. | 2015 | {#192} | Exclusion criteria (d) |
| Lindgren, M., et al. | 2020 | {#193} | Exclusion criteria (d) |
| Linszen, D. H., et al. | 1997 | {#194} | Exclusion criteria (d) |
| Liu, C. C., et al. | 2011 | {#195} | Exclusion criteria (d) |
| Lopez-Diaz, A., et al. | 2020 | {#244} | Exclusion criteria (d) |
| Lyne, J., et al. | 2012 | {#196} | Exclusion criteria (d) |
| Madero, S., et al. | 2020 | {#102} | Exclusion criteria (e) |
| Malla, A., et al. | 2006 | {#103} | Exclusion criteria (e) |
| Mandarelli, G., et al. | 2017 | {#104} | Exclusion criteria (e) |
| Manrique-Garcia, E., et al. | 2012 | {#197} | Exclusion criteria (d) |
| Marchira, C. R., et al. | 2019 | {#105} | Exclusion criteria (e) |
| Marneros, A., et al. | 2003 | {#246} | Overlapping dataset |
| Marneros, A., et al. | 2002 | {#247} | Overlapping dataset |
| Marneros, A., et al. | 2003 | {#248} | Overlapping dataset |
| Marneros, A., et al. | 2003 | {#246} | Exclusion criteria (d) |
| Marneros, A., et al. | 2005 | {#198} | Exclusion criteria (d) |
| Maurer, K., et al. | 2018 | {#106} | Exclusion criteria (e) |
| Maximo, J. O., et al. | 2020 | {#230} | Exclusion criteria (d) |
| McGorry, P. D., et al. | 2017 | {#107} | Exclusion criteria (e) |
| McHugh, M. J., et al. | 2018 | {#261} | Exclusion criteria (d) |
| Meneghelli, A., et al. | 2020 | {#108} | Exclusion criteria (e) |
| Miklowitz, D. J., et al. | 2014 | {#199} | Exclusion criteria (d) |
| Minichino, A., et al. | 2019 | {#200} | Exclusion criteria (d) |
| Mojtabai, R., et al. | 2000 | {#201} | Exclusion criteria (d) |
| Montemagni, C., et al. | 2015 | {#202} | Exclusion criteria (d) |
| Montgomery, W., et al. | 2015 | {#109} | Exclusion criteria (e) |
| Morgan, K., et al. | 2011 | {#110} | Exclusion criteria (e) |
| Nash, L., et al. | 2004 | {#111} | Exclusion criteria (e) |
| Nelson, B., et al. | 2013 | {#234} | Overlapping dataset |
| Noordsy, D. L., et al. | 2001 | {#112} | Exclusion criteria (e) |
| Palma-Sevillano, C., et al. | 2011 | {#113} | Exclusion criteria (e) |
| Pelizza, L., et al. | 2020 | {#277} | Exclusion criteria (d) |
| Pelletier, A. L., et al. | 2013 | {#203} | Exclusion criteria (d) |
| Pillmann, F., et al. | 2001 | {#249} | Overlapping dataset |
| Pillmann, F., et al. | 2002 | {#250} | Overlapping dataset |
| Pillmann, F., et al. | 2003 | {#251} | Overlapping dataset |
| Pillmann, F., et al. | 2002 | {#252} | Overlapping dataset |
| Pillmann, F., et al. | 2005 | {#253} | Overlapping dataset |
| Pillmann, F., et al. | 2012 | {#77} | Overlapping dataset |
| Polari, A., et al. | 2013 | {#204} | Exclusion criteria (d) |
| Poloni, N., et al. | 2018 | {#114} | Exclusion criteria (e) |
| Potkin, S. G., et al. | 2001 | {#115} | Exclusion criteria (e) |
| Poulin, J., et al. | 2003 | {#116} | Exclusion criteria (e) |
| Power, P., et al. | 1998 | {#205} | Exclusion criteria (d) |
| Preston, N. J., et al. | 2003 | {#117} | Exclusion criteria (e) |
| Preti, A., et al. | 2009 | {#118} | Exclusion criteria (e) |
| Pruessner, M., et al. | 2011 | {#231} | Exclusion criteria (d) |
| Pruessner, M., et al. | 2019 | {#119} | Exclusion criteria (e) |
| Rapoport, J. L., et al. | 1997 | {#120} | Exclusion criteria (e) |
| Rasmussen, S. A., et al. | 2016 | {#121} | Exclusion criteria (e) |
| Rasmussen, S. A., et al. | 2017 | {#122} | Exclusion criteria (e) |
| Rausch, F., et al. | 2013 | {#206} | Exclusion criteria (d) |
| Reay, R., et al. | 2010 | {#207} | Exclusion criteria (d) |
| Remberk, B., et al. | 2018 | {#123} | Exclusion criteria (e) |
| Roe, D., et al. | 2011 | {#124} | Exclusion criteria (e) |
| Sahin, S., et al. | 2013 | {#208} | Exclusion criteria (d) |
| Salem, M. O., et al. | 2009 | {#59} | Exclusion criteria (d) |
| Salvatore, P., et al. | 2014 | {#278} | Exclusion criteria (d) |
| Sanger, T. M., et al. | 1999 | {#125} | Exclusion criteria (e) |
| Sankaranarayanan, A., et al. | 2016 | {#126} | Exclusion criteria (e) |
| Sarpal, D. K., et al. | 2015 | {#127} | Exclusion criteria (e) |
| Satghare, P., et al. | 2020 | {#268} | Exclusion criteria (d) |
| Schimmelmann, B. G., et al. | 2015 | {#209} | Exclusion criteria (d) |
| Schmidt, A., et al. | 2014 | {#210} | Exclusion criteria (d) |
| Schroeder, K., et al. | 2013 | {#211} | Exclusion criteria (d) |
| Schultze-Lutter, F., et al. | 2017 | {#212} | Exclusion criteria (d) |
| Schultze-Lutter, F., et al. | 2020 | {#213} | Exclusion criteria (d) |
| Severance, E. G., et al. | 2015 | {#214} | Exclusion criteria (d) |
| Sigrunarson, V., et al. | 2017 | {#128} | Exclusion criteria (e) |
| Sikich, L., et al. | 2004 | {#129} | Exclusion criteria (e) |
| Silverstein, S., et al. | 2006 | {#279} | Exclusion criteria (d) |
| Sirota, P., et al. | 1999 | {#280} | Exclusion criteria (d) |
| Soyata, A. Z., et al. | 2018 | {#274} | Exclusion criteria (d) |
| Spidel, A., et al. | 2015 | {#130} | Exclusion criteria (e) |
| Stralin, P., et al. | 2019 | {#217} | Exclusion criteria (d) |
| Studerus, E., et al. | 2020 | {#218} | Exclusion criteria (d) |
| Su, W. J., et al. | 2015 | {#131} | Exclusion criteria (e) |
| Subotnik, K. L., et al. | 2000 | {#132} | Exclusion criteria (e) |
| Susser, E., et al. | 1998 | {#133} | Exclusion criteria (e) |
| Svindseth, M. F., et al. | 2010 | {#134} | Exclusion criteria (e) |
| Swett, C., et al. | 1997 | {#135} | Exclusion criteria (e) |
| Taiminen, T., et al. | 2000 | {#136} | Exclusion criteria (e) |
| Tan, C. Z., et al. | 2019 | {#219} | Exclusion criteria (d) |
| Tor, J., et al. | 2020 | {#232} | Exclusion criteria (d) |
| Tsujino, N., et al. | 2018 | {#220} | Exclusion criteria (d) |
| Turner, M., et al. | 2006 | {#221} | Exclusion criteria (d) |
| Ucok, A., et al. | 2006 | {#137} | Exclusion criteria (e) |
| Ucok, A., et al. | 2011 | {#138} | Exclusion criteria (e) |
| Udomratn, P., et al. | 2012 | {#146} | Exclusion criteria (a) |
| Valevski, A., et al. | 2012 | {#139} | Exclusion criteria (e) |
| Vazquez-Bourgon, J., et al. | 2010 | {#222} | Exclusion criteria (d) |
| Ventura, J., et al. | 2021 | {#140} | Exclusion criteria (e) |
| Ventura, J., et al. | 2004 | {#141} | Exclusion criteria (e) |
| Vernal, D. L., et al. | 2015 | {#233} | Exclusion criteria (d) |
| Yamada, A. M., et al. | 2010 | {#142} | Exclusion criteria (e) |
| Zanello, A., et al. | 2017 | {#143} | Exclusion criteria (e) |
| Zheng, S. S., et al. | 2015 | {#223} | Exclusion criteria (d) |

**eLimitations**

This study has some limitations. We did not search for grey literature; however, the primary outcome was non-comparative in nature. Also, we did not include comparison groups of patients with first-episode schizophrenia or affective psychoses; however, pertinent studies may be hardly reported. We uncovered high heterogeneity; we sought to partially explain it via meta-regression analyses, which indicated, for example that higher quality standard in conducting studies in this patient population might detect higher rates of psychotic recurrence (Lopez-Diaz *et al.*, 2020). These meta-regression analyses are the largest one conducted to date in short-lived psychotic episodes. Finally, some meta-analyses (e.g. APPD without symptoms of schizophrenia) were conducted on a small number of studies; future research is needed to confirm these initial findings

REFERENCES

**Aadamsoo K, Saluveer E, Kueuenarpuu H, Vasar V and Maron E**. (2011) Diagnostic stability over 2 years in patients with acute and transient psychotic disorders. *Nordic Journal of Psychiatry* **65**: 381-388.

**Abe T, Otsuka K and Kato S**. (2006) Long-term clinical course of patients with acute polymorphic psychotic disorder without symptoms of schizophrenia. *Psychiatry Clin Neurosci* **60**: 452-457.

**Addington J, Chaves A and Addington D**. (2006) Diagnostic stability over one year in first-episode psychosis. *Schizophrenia Research* **86**: 71-75.

**Addington J, Liu L, Buchy L, Cadenhead KS, Cannon TD, Cornblatt BA, Perkins DO, Seidman LJ, Tsuang MT, Walker EF, Woods SW, Bearden CE, Mathalon DH and McGlashan TH**. (2015) North American Prodrome Longitudinal Study (NAPLS 2) The Prodromal Symptoms. *Journal of Nervous and Mental Disease* **203**: 328-335.

**Amini H, Alaghband-rad J, Omid A, Sharifi V, Davari-Ashtiani R, Momeni F and Aminipour Z**. (2005) Diagnostic stability in patients with first-episode psychosis. *Australasian psychiatry : bulletin of Royal Australian and New Zealand College of Psychiatrists* **13**: 388-392.

**Armando M, Pontillo M, De Crescenzo F, Mazzone L, Monducci E, Lo Cascio N, Santonastaso O, Pucciarini ML, Vicari S, Schimmelmann BG and Schultze-Lutter F**. (2015) Twelve-month psychosis-predictive value of the ultra-high risk criteria in children and adolescents. *Schizophrenia Research* **169**: 186-192.

**Baldwin P, Browne D, Scully PJ, Quinn JF, Morgan MG, Kinsella A, Owens JM, Russell V, O'Callaghan E and Waddington JL**. (2005) BIBLIO Epidemiology of first-episode psychosis: illustrating the challenges across diagnostic boundaries through the Cavan-Monaghan study at 8 years. *Schizophr Bull* **31**: 624-638.

**Barak Y, Levy D, Szor H and Aizenberg D**. (2011) First-onset functional brief psychoses in the elderly. *Canadian geriatrics journal : CGJ* **14**: 30-33.

**Bjorkenstam E, Bjorkenstam C, Hjern A, Reutfors J and Boden R**. (2013) A five year diagnostic follow-up of 1840 patients after a first episode non-schizophrenia and non-affective psychosis. *Schizophrenia Research* **150**: 205-210.

**Caseiro O, Perez-Iglesias R, Mata I, Martinez-Garcia O, Maria Pelayo-Teran J, Tabares-Seisdedos R, Ortiz-Garcia de la Foz V, Vazquez-Barquero JL and Crespo-Facorro B**. (2012) Predicting relapse after a first episode of non-affective psychosis: A three-year follow-up study. *Journal of Psychiatric Research* **46**: 1099-1105.

**Castagnini A, Bertelsen A and Berrios GE**. (2008) Incidence and diagnostic stability of ICD-10 acute and transient psychotic disorders. *Comprehensive Psychiatry* **49**: 255-261.

**Castagnini A and Foldager L**. (2014) Epidemiology, Course and Outcome of Acute Polymorphic Psychotic Disorder: Implications for ICD-11. *Psychopathology* **47**: 202-206.

**Castagnini AC, Munk-Jorgensen P and Bertelsen A**. (2016) Short-term course and outcome of acute and transient psychotic disorders: Differences from other types of psychosis with acute onset. *International Journal of Social Psychiatry* **62**: 51-56.

**Castro-Fornieles J, Baeza I, de la Serna E, Gonzalez-Pinto A, Parellada M, Graell M, Moreno D, Otero S and Arango C**. (2011) Two-year diagnostic stability in early-onset first-episode psychosis. *Journal of Child Psychology and Psychiatry* **52**: 1089-1098.

**Chang WC, Pang SLK, Chung DWS and Chan SSM**. (2009) Five-year stability of ICD-10 diagnoses among Chinese patients presented with first-episode psychosis in Hong Kong. *Schizophrenia Research* **115**: 351-357.

**Chen EYH, Hui CLM, Lam MML, Chiu CPY, Law CW, Chung DWS, Tso S, Pang EPF, Chan KT, Wong YC, Mo FYM, Chan KPM, Yao TJ, Hung SF and Honer WG**. (2010) Maintenance treatment with quetiapine versus discontinuation after one year of treatment in patients with remitted first episode psychosis: randomised controlled trial. *British Medical Journal* **341**.

**Correll CU, Lencz T, Smith CW, Auther AM, Nakayama EY, Hovey L, Olsen R, Shah M, Foley C and Cornblatt BA**. (2005) Prospective study of adolescents with subsyndromal psychosis: Characteristics and outcome. *Journal of Child and Adolescent Psychopharmacology* **15**: 418-433.

**Das SK, Malhotra S and Basu D**. (1999) Family study of acute and transient psychotic disorders: comparison with schizophrenia. *Social Psychiatry and Psychiatric Epidemiology* **34**: 328-332.

**Dragt S, Nieman DH, Veltman D, Becker HE, van de Fliert R, de Haan L and Linszen DH**. (2011) Environmental factors and social adjustment as predictors of a first psychosis in subjects at ultra high risk. *Schizophr Res* **125**: 69-76.

**Esan O and Fawole O**. (2013) Comparison of the Profile of Patients With Acute and Transient Psychotic Disorder and Schizophrenia in a Nigerian Teaching Hospital. *Journal of Neuropsychiatry and Clinical Neurosciences* **25**: 327-334.

**Fraguas D, de Castro MJ, Medina O, Parellada M, Moreno D, Graell M, Merchan-Naranjo J and Arango C**. (2008) Does diagnostic classification of early-onset psychosis change over follow-up? *Child Psychiatry & Human Development* **39**: 137-145.

**Fusar-Poli P, Cappucciati M, De Micheli A, Rutigliano G, Bonoldi I, Tognin S, Ramella-Cravaro V, Castagnini A and McGuire P**. (2017) Diagnostic and Prognostic Significance of Brief Limited Intermittent Psychotic Symptoms (BLIPS) in Individuals at Ultra High Risk. *Schizophrenia Bulletin* **43**: 48-56.

**Fusar-Poli P, De Micheli A, Chalambrides M, Singh A, Augusto C and McGuire P**. (2019) Unmet needs for treatment in 102 individuals with brief and limited intermittent psychotic symptoms (BLIPS): implications for current clinical recommendations. *Epidemiology and psychiatric sciences* **29**: e67-e67.

**Fusar-Poli P, Hobson R, Raduelli M and Balottin U**. (2012) Reliability and Validity of the Comprehensive Assessment of the at Risk Mental State, Italian Version (CAARMS-I). *Current Pharmaceutical Design* **18**: 386-391.

**Fusar-Poli P, Nelson B, Valmaggia L, Yung AR and McGuire PK**. (2014) Comorbid Depressive and Anxiety Disorders in 509 Individuals With an At-Risk Mental State: Impact on Psychopathology and Transition to Psychosis. *Schizophrenia Bulletin* **40**: 120-131.

**Haahr U, Friis S, Larsen TK, Melle I, Johannessen JO, Opjordsmoen S, Simonsen E, Rund BR, Vaglum P and McGlashan T**. (2008) First-episode psychosis: Diagnostic stability over one and two years. *Psychopathology* **41**: 322-329.

**Heslin M, Lomas B, Lappin JM, Donoghue K, Reininghaus U, Onyejiaka A, Croudace T, Jones PB, Murray RM, Fearon P, Dazzan P, Morgan C and Doody GA**. (2015) Diagnostic change 10 years after a first episode of psychosis. *Psychological Medicine* **45**: 2757-2769.

**Hollis C**. (2000) Adult outcomes of child- and adolescent-onset schizophrenia: diagnostic stability and predictive validity. *Am J Psychiatry* **157**: 1652-1659.

**Huguelet P, Schneider El Gueddari N and Glauser D**. (2001) Stability of DSM-III-R diagnoses: study of a case register. *Psychopathology* **34**: 118-122.

**Jager M, Hintermayr M, Bottlender R, Strauss A and Moller HJ**. (2003) Course and outcome of first-admitted patients with acute and transient psychotic disorders (ICD-10 : F23) - Focus on relapses and social adjustment. *European Archives of Psychiatry and Clinical Neuroscience* **253**: 209-215.

**Jørgensen P**. (1995) Comparative outcome of first-admission patients with delusional beliefs. *Eur Psychiatry* **10**: 276-281.

**Jørgensen P, Bennedsen B, Christensen J and Hyllested A**. (1997) Acute and transient psychotic disorder: a 1-year follow-up study. *Acta Psychiatr Scand* **96**: 150-154.

**Kar SK and Dhanasekaran S**. (2017) Outcome of acute and transient psychotic disorder in an index episode: A study from a tertiary care centre in North India. *Asian J Psychiatr* **25**: 101-105.

**Katsura M, Ohmuro N, Obara C, Kikuchi T, Ito F, Miyakoshi T, Matsuoka H and Matsumoto K**. (2014) A naturalistic longitudinal study of at-risk mental state with a 2.4 year follow-up at a specialized clinic setting in Japan. *Schizophrenia Research* **158**: 32-38.

**Kéri S, Kiss I and Kelemen O**. (2009) Effects of a neuregulin 1 variant on conversion to schizophrenia and schizophreniform disorder in people at high risk for psychosis. *Mol Psychiatry* **14**: 118-119.

**Kim E, Jang JH, Park H-Y, Shim G, Hwang JY, Kim SN and Kwon JS**. (2012) Pharmacotherapy and clinical characteristics of ultra-high-risk for psychosis according to conversion status: a naturalistic observational study. *Early Intervention in Psychiatry* **6**: 30-37.

**Kim JS, Baek JH, Choi JS, Lee D, Kwon JS and Hong KS**. (2011) Diagnostic stability of first-episode psychosis and predictors of diagnostic shift from non-affective psychosis to bipolar disorder: A retrospective evaluation after recurrence. *Psychiatry Research* **188**: 29-33.

**Kingston T, Scully PJ, Browne DJ, Baldwin PA, Kinsella A, Russell V, O'Callaghan E and Waddington JL**. (2013) Diagnostic trajectory, interplay and convergence/divergence across all 12 DSM-IV psychotic diagnoses: 6-year follow-up of the Cavan-Monaghan First Episode Psychosis Study (CAMFEPS). *Psychological Medicine* **43**: 2523-2533.

**Koike S, Takano Y, Iwashiro N, Satomura Y, Suga M, Nagai T, Natsubori T, Tada M, Nishimura Y, Yamasaki S, Takizawa R, Yahata N, Araki T, Yamasue H and Kasai K**. (2013) A multimodal approach to investigate biomarkers for psychosis in a clinical setting: The integrative neuroimaging studies in schizophrenia targeting for early intervention and prevention (IN-STEP) project. *Schizophrenia Research* **143**: 116-124.

**Kotlicka-Antczak M, Pawelczyk T, Rabe-Jablonska J and Pawelczyk A**. (2015) PORT (Programme of Recognition and Therapy): the first Polish recognition and treatment programme for patients with an at-risk mental state. *Early Intervention in Psychiatry* **9**: 339-342.

**Koutsouleris N, Meisenzahl EM, Davatzikos C, Bottlender R, Frodl T, Scheuerecker J, Schmitt G, Zetzsche T, Decker P, Reiser M, Moller HJ and Gaser C**. (2009) Use of neuroanatomical pattern classification to identify subjects in at-risk mental states of psychosis and predict disease transition. *Arch Gen Psychiatry* **66**: 700-712.

**Labad J, Stojanovic-Perez A, Montalvo I, Sole M, Cabezas A, Ortega L, Moreno I, Vilella E, Martorell L, Reynolds RM and Gutierrez-Zotes A**. (2015) Stress biomarkers as predictors of transition to psychosis in at-risk mental states: Roles for cortisol, prolactin and albumin. *Journal of Psychiatric Research* **60**: 163-169.

**Lam MML, Hung SF and Chen EYH**. (2006) Transition to psychosis: 6-month follow-up of a Chinese high-risk group in Hong Kong. *Australian and New Zealand Journal of Psychiatry* **40**: 414-420.

**Langbein K, Schmidt U, Schack S, Biesel NJ, Rudzok M, Amminger GP, Berger M, Sauer H and Smesny S**. (2018) State marker properties of niacin skin sensitivity in ultra-high risk groups for psychosis - An optical reflection spectroscopy study. *Schizophrenia Research* **192**: 377-384.

**Lee J, Rekhi G, Mitter N, Bong YL, Kraus MS, Lam M, Rapisarda A, Lee T-S, Subramaniam M, Chong SA and Keefe RSE**. (2013) The Longitudinal Youth at Risk Study (LYRIKS) - An Asian UHR perspective. *Schizophrenia Research* **151**: 279-283.

**Lee TY, Kim SN, Correll CU, Byun MS, Kim E, Jang JH, Kang D-H, Yun J-Y and Kwon JS**. (2014) Symptomatic and functional remission of subjects at clinical high risk for psychosis: A 2-year naturalistic observational study. *Schizophrenia Research* **156**: 266-271.

**Lemos-Giraldez S, Vallina-Fernandez O, Fernandez-Iglesias P, Vallejo-Seco G, Fonseca-Pedrero E, Paino-Pineiro M, Sierra-Baigrie S, Garcia-Pelayo P, Pedrejon-Molino C, Alonso-Bada S, Gutierrez-Perez A and Angel Ortega-Ferrandez J**. (2009) Symptomatic and functional outcome in youth at ultra-high risk for psychosis: A longitudinal study. *Schizophrenia Research* **115**: 121-129.

**Lopez-Diaz A, Fernandez-Genzalez JL, Lara I, Crespo-Facorro B and Ruiz-Veguilla M**. (2019) The prognostic role of catatonia, hallucinations, and symptoms of schizophrenia in acute and transient psychosis. *Acta Psychiatrica Scandinavica* **140**: 574-585.

**Lopez-Diaz A, Fernandez-Gonzalez JL, Lara I and Ruiz-Veguilla M**. (2020) Predictors of diagnostic stability in acute and transient psychotic disorders: validation of previous findings and implications for ICD-11. *European Archives of Psychiatry and Clinical Neuroscience* **270**: 291-299.

**Lopez-Diaz A, Lorenzo-Herrero P, Lara I, Luis Fernandez-Gonzalez J and Ruiz-Veguilla M**. (2018) Acute stress and substance use as predictors of suicidal behaviour in acute and transient psychotic disorders. *Psychiatry Research* **269**: 414-418.

**Lyne J, O'Donoghue B, Owens E, Renwick L, Madigan K, Kinsella A, Clarke M, Turner N and O'Callaghan E**. (2012) Prevalence of item level negative symptoms in first episode psychosis diagnoses. *Schizophrenia Research* **135**: 128-133.

**Mason O, Startup M, Halpin S, Schall U, Conrad A and Carr V**. (2004) Risk factors for transition to first episode psychosis among individuals with 'at-risk mental states'. *Schizophrenia Research* **71**: 227-237.

**Matsumoto K, Katsura M, Tsujino N, Nishiyama S, Nemoto T, Katagiri N, Takahashi T, Higuchi Y, Ohmuro N, Matsuoka H, Suzuki M and Mizuno M**. (2019) Federated multi-site longitudinal study of at-risk mental state for psychosis in Japan. *Schizophrenia Research* **204**: 343-352.

**Metzler S, Dvorsky D, Wyss C, Mueller M, Traber-Walker N, Walitza S, Theodoridou A, Roessler W and Heekeren K**. (2014) Neurocognitive profiles in help-seeking individuals: comparison of risk for psychosis and bipolar disorder criteria. *Psychological Medicine* **44**: 3543-3555.

**Moller HJ, Jager M, Riedel M, Obermeier M, Strauss A and Bottlender R**. (2011) The Munich 15-year follow-up study (MUFUSSAD) on first-hospitalized patients with schizophrenic or affective disorders: Assessing courses, types and time stability of diagnostic classification. *European Psychiatry* **26**: 231-243.

**Narayanaswamy JC, Shanmugam VH, Raveendranathan D, Viswanath B and Muralidharan K**. (2012) Short-term diagnostic stability of acute psychosis: data from a tertiary care psychiatric center in South India. *Indian journal of psychological medicine* **34**: 176-178.

**Nelson B, Yuen K and Yung AR**. (2011) Ultra high risk (UHR) for psychosis criteria: Are there different levels of risk for transition to psychosis? *Schizophrenia Research* **125**: 62-68.

**Nieman DH, Velthorst E, Becker HE, de Haan L, Dingemans PM, Linszen DH, Birchwood M, Patterson P, Salokangas RK, Heinimaa M, Heinz A, Juckel G, von Reventlow HG, Morrison A, Schultze-Lutter F, Klosterkötter J, Ruhrmann S and group E**. (2013) The Strauss and Carpenter Prognostic Scale in subjects clinically at high risk of psychosis. *Acta Psychiatr Scand* **127**: 53-61.

**Okasha A, Eldawla AS, Khalil AH and Saad A**. (1993) PRESENTATION OF ACUTE-PSYCHOSIS IN AN EGYPTIAN SAMPLE - A TRANSCULTURAL COMPARISON. *Comprehensive Psychiatry* **34**: 4-9.

**Pedros A, Marti J, Gutierrez G, Tenias JM and Ruescas S**. (2009) Two-year diagnostic stability and prognosis in acute psychotic episodes. *Actas Espanolas De Psiquiatria* **37**: 245-251.

**Peralta D, Studerus E, Andreou C, Beck K, Ittig S, Leanza L, Egloff L and Riecher-Rossler A**. (2019) Exploring the predictive power of the unspecific risk category of the Basel Screening Instrument for Psychosis. *Early Intervention in Psychiatry* **13**: 969-976.

**Pillmann F, Wustmann T and Marneros A**. (2012) Acute and transient psychotic disorders versus persistent delusional disorders: a comparative longitudinal study. *Psychiatry Clin Neurosci* **66**: 44-52.

**Poon JYK and Leung CM**. (2017) Outcome of first-episode acute and transient psychotic disorder in Hong Kong Chinese: a 20-year retrospective follow-up study. *Nordic Journal of Psychiatry* **71**: 139-144.

**Queirazza F, Semple DM and Lawrie SM**. (2014) Transition to schizophrenia in acute and transient psychotic disorders. *British Journal of Psychiatry* **204**: 299-305.

**Rahm C and Cullberg J**. (2007) Diagnostic stability over 3 years in a total group of first-episode psychosis patients. *Nord J Psychiatry* **61**: 189-193.

**Rajkumar RP**. (2015) Recurrent acute and transient psychotic disorder: A pilot study. *Asian Journal of Psychiatry* **14**: 61-64.

**Ranjan S, Shakya R and Shyangwa P**. (2014) Diagnostic Stability of Acute and Transient Psychotic Disorders in Patients Attending Tertiary Care Hospital. *Journal of Universal College of Medical Sciences* **2**.

**Remberk B, Bazynska AK, Krempa-Kowalewska A and Rybakowski F**. (2014) Adolescent insanity revisited: Course and outcome in early-onset schizophrenia spectrum psychoses in an 8-year follow-up study. *Comprehensive Psychiatry* **55**: 1174-1181.

**Riecher-Rossler A, Pflueger MO, Aston J, Borgwardt SJ, Brewer WJ, Gschwandtner U and Stieglitz RD**. (2009) Efficacy of Using Cognitive Status in Predicting Psychosis: A 7-Year Follow-Up. *Biological Psychiatry* **66**: 1023-1030.

**Rufino A, Uchida RR, Vilela JAA, Marques JMA, Zuardi AW and Del-Ben CM**. (2005) Stability of the diagnosis of first-episode psychosis made in an emergency setting. *General Hospital Psychiatry* **27**: 189-193.

**Rusaka M and Rancans E**. (2014a) First-episode acute and transient psychotic disorder in Latvia: A 6-year follow-up study. *Nordic Journal of Psychiatry* **68**: 24-29.

**Rusaka M and Rancans E**. (2014b) A prospective follow-up study of first-episode acute transient psychotic disorder in Latvia. *Annals of General Psychiatry* **13**.

**Rutigliano G, Merlino S, Minichino A, Patel R, Davies C, Oliver D, De Micheli A, McGuire P and Fusar-Poli P**. (2018) Long term outcomes of acute and transient psychotic disorders: The missed opportunity of preventive interventions. *European Psychiatry* **52**: 126-133.

**Sajith SG, Chandrasekaran R, Unni KES and Sahai A**. (2002) Acute polymorphic psychotic disorder: diagnostic stability over 3 years. *Acta Psychiatrica Scandinavica* **105**: 104-109.

**Salem MO, Moselhy HF, Attia H and Yousef S**. (2009) Psychogenic Psychosis Revisited: A Follow up Study. *International journal of health sciences* **3**: 45-49.

**Salvatore P, Baldessarini RJ, Tohen M, Khalsa H-MK, Sanchez-Toledo JP, Zarate CA, Jr., Vieta E and Maggini C**. (2009) McLean-Harvard International First-Episode Project: Two-Year Stability of DSM-IV Diagnoses in 500 First-Episode Psychotic Disorder Patients. *Journal of Clinical Psychiatry* **70**: 458-466.

**Salvatore P, Baldessarini RJ, Tohen M, Khalsa HM, Sanchez-Toledo JP, Zarate CA, Jr., Vieta E and Maggini C**. (2011) McLean-Harvard International First-Episode Project: two-year stability of ICD-10 diagnoses in 500 first-episode psychotic disorder patients. *J Clin Psychiatry* **72**: 183-193.

**Schimmelmann BG, Conus P, Edwards J, McGorry PD and Lambert M**. (2005) Diagnostic stability 18 months after treatment initiation for first-episode psychosis. *Journal of Clinical Psychiatry* **66**: 1239-1246.

**Schultze-Lutter F, Klosterkötter J and Ruhrmann S**. (2014) Improving the clinical prediction of psychosis by combining ultra-high risk criteria and cognitive basic symptoms. *Schizophr Res* **154**: 100-106.

**Schwartz JE, Fennig S, Tanenberg-Karant M, Carlson G, Craig T, Galambos N, Lavelle J and Bromet EJ**. (2000) Congruence of diagnoses 2 years after a first-admission diagnosis of psychosis. *Archives of General Psychiatry* **57**: 593-600.

**Simon AE, Graedel M, Cattapan-Ludewig K, Gruber K, Ballinari P, Roth B and Umbricht D**. (2012) Cognitive functioning in at-risk mental states for psychosis and 2-year clinical outcome. *Schizophrenia Research* **142**: 108-115.

**Singh SP, Burns T, Amin S, Jones PB and Harrison G**. (2004) Acute and transient psychotic disorders: precursors, epidemiology, course and outcome. *British Journal of Psychiatry* **185**: 452-459.

**Spada G, Molteni S, Pistone C, Chiappedi M, McGuire P, Fusar-Poli P and Balottin U**. (2016) Identifying children and adolescents at ultra high risk of psychosis in Italian neuropsychiatry services: a feasibility study. *European Child & Adolescent Psychiatry* **25**: 91-106.

**Subramaniam M, Pek E, Verma S, Chan YH and Chong SA**. (2007) Diagnostic stability 2 years after treatment initiation in the Early Psychosis Intervention Programme in Singapore. *Australian and New Zealand Journal of Psychiatry* **41**: 495-500.

**Suda K, Hayashi N and Hiraga M**. (2005) Predicting features of later development of schizophrenia among patients with acute and transient psychotic disorder. *Psychiatry Clin Neurosci* **59**: 146-150.

**Thangadurai P, Gopalakrishnan R, Kurian S and Jacob KS**. (2006) Diagnostic stability and status of acute and transient psychotic disorders. *Br J Psychiatry* **188**: 293.

**van Tricht MJ, Nieman DH, Koelman JH, van der Meer JN, Bour LJ, de Haan L and Linszen DH**. (2010) Reduced parietal P300 amplitude is associated with an increased risk for a first psychotic episode. *Biol Psychiatry* **68**: 642-648.

**Veen ND, Selten JP, Van der Tweel I, Feller WG, Hoek HW and Kahn RS**. (2004) Cannabis use and age at onset of schizophrenia. *American Journal of Psychiatry* **161**: 501-506.

**Wang H-y, Guo W-j, Li X-j, Tao Y-j, Meng Y-j, Wang Q, Deng W and Li T**. (2018) Higher required dosage of antipsychotics to relieve the symptoms of first-onset Acute and Transient Psychotic Disorder (ATPD) predicted the subsequent diagnostic transition to schizophrenia: A longitudinal study. *Schizophrenia Research* **193**: 461-462.

**Woods SW, Addington J, Cadenhead KS, Cannon TD, Cornblatt BA, Heinssen R, Perkins DO, Seidman LJ, Tsuang MT, Walker EF and McGlashan TH**. (2009) Validity of the Prodromal Risk Syndrome for First Psychosis: Findings From the North American Prodrome Longitudinal Study. *Schizophrenia Bulletin* **35**: 894-908.

**Zhang T, Li H, Woodberry KA, Seidman LJ, Zheng L, Li H, Zhao S, Tang Y, Guo Q, Lu X, Zhuo K, Qian Z, Chow A, Li C, Jiang K, Xiao Z and Wang J**. (2014) Prodromal psychosis detection in a counseling center population in China: An epidemiological and clinical study. *Schizophrenia Research* **152**: 391-399.

**Ziermans TB, Schothorst PF, Sprong M and van Engeland H**. (2011) Transition and remission in adolescents at ultra-high risk for psychosis. *Schizophr Res* **126**: 58-64.
